# Guideline for the Comprehensive Perioperative Management of Older Patients With Lung Cancer (2025)

**DOI:** 10.1111/jebm.70178

**Published:** 2026-08-11

**Authors:** Daiping Li, Rui Liang, Birong Dong, Lunxu Liu, Ning Ge, National Clinical Research Center for Geriatrics, West China Hospital of Sichuan University

**Affiliations:** ^1^ Department of Geriatrics and National Clinical Research Center for Geriatrics West China Hospital of Sichuan University Chengdu China; ^2^ Department of Thoracic Surgery West China Hospital, Sichuan University Chengdu China

**Keywords:** comprehensive management, guideline, lung cancer, older adult, perioperative period

## Abstract

With the accelerating population aging, the number of older adult patients with lung cancer continues to increase. These patients often present with multiple chronic diseases and geriatric syndromes, resulting in more complex perioperative management and significantly increased surgical risks. This guideline was developed and reported on the basis of the Grading of Recommendations Assessment, Development and Evaluation (GRADE) system and the Reporting Items for Practice Guidelines in Healthcare (RIGHT) checklist. Focusing on 28 key clinical issues commonly encountered among older adults with lung cancer, such as frailty, malnutrition, and falls, a total of 55 recommendations were formulated. This guideline aims to provide standardized perioperative management strategies through comprehensive geriatric assessment, comorbidity management, and early identification and intervention for complications to reduce the incidence of postoperative complications and improve patients’ quality of life.

## 1 Introduction

With the acceleration of population aging, the number of older patients with lung cancer in China continues to rise. These patients often present with multiple chronic conditions and geriatric syndromes, which complicate perioperative management and significantly increase surgical risks [1]. Optimizing perioperative management strategies to effectively reduce postoperative complications and improve patients’ quality of life has become a critical issue requiring urgent attention in both geriatric medicine and thoracic surgery. Geriatric syndromes, such as frailty and malnutrition, are prevalent among older patients with lung cancer and are associated with increased risk of complications, prolonged hospital stays, delayed functional recovery, and poorer overall prognosis [2]. In addition, older patients frequently have comorbidities such as cardiovascular disease, further increasing the risk of perioperative mortality and complications [3, 4]. Postoperative complications, including infection, respiratory failure, cardiac dysfunction, and delirium, are more common in older patients. Therefore, early identification of complication risks and implementation of targeted interventions are crucial for improving postoperative recovery outcomes and shortening hospital stays [1]. This guideline provides specific recommendations on the screening and intervention for geriatric syndromes, management of comorbidities, and prevention and treatment of complications. Through standardized and comprehensive management approaches, it aims to improve both survival and quality of life, thereby providing more scientific and high‐quality medical care to older patients with lung cancer [1].

## 2 Methods

This guideline was developed in accordance with the procedures outlined in the WHO Handbook for Guideline Development, the Guiding Principles for the Development/Revision of Clinical Practice Guidelines in China (2022 Edition), and the Appraisal of Guidelines for Research and Evaluation II (AGREE II) instrument [5]. The guideline was reported in accordance with the Reporting Items for Practice Guidelines in Healthcare (RIGHT) statement [6, 7].

### 2.1 Initiating Organization and Registration of the Guideline

This guideline was jointly developed by 94 multidisciplinary experts from 26 institutions, organized by West China Hospital of Sichuan University. The guideline was registered on the International Practice Guideline Registration for Transparency (PREPARE‐2025CN579), and its protocol has been published in the *Chinese Journal of Evidence‐Based Medicine* [1].

### 2.2 Guideline Development Working Group

Five working groups were established, including the Steering Committee, the Secretariat Group, the Evidence Evaluation Group, the Consensus Expert Group, and the External Review Expert Group (Supporting information A). The Steering Committee consisted of three experts in geriatrics, lung cancer, and evidence‐based medicine, responsible for defining working group roles and the scope of the guideline, reviewing conflicts of interest, validating clinical questions and evidence quality, organizing consensus meetings, overseeing the process, and approving final recommendations as well as their dissemination and updates. The Secretariat Group consisted of three researchers in geriatrics and guideline methodology, primarily responsible for guideline registration and protocol development, collecting clinical questions and outcome measures, organizing and documenting all process files, and coordinating related meetings. The Evidence Evaluation Group consisted of 42 multidisciplinary experts from geriatrics, thoracic surgery, pulmonology, anesthesiology, rehabilitation medicine, clinical nutrition, and the evidence‐based medicine center and is responsible for population, intervention, comparison, and outcome (PICO) deconstruction of clinical questions, literature search and screening, evidence quality assessment, Grading of Recommendations Assessment, Development and Evaluation (GRADE) evidence grading, drafting evidence summary tables, preliminary formulation of recommendations, and writing the first draft of the guideline. The Consensus Expert Group consisted of 41 multidisciplinary experts from geriatrics, pulmonology, anesthesiology, rehabilitation medicine, clinical nutrition, and the evidence‐based medicine center, responsible for the Delphi consultation on clinical questions and outcome measures, guiding evidence evaluation and grading, reaching consensus on recommendations, and overseeing the revision and improvement of the guideline. The External Review Expert Group consisted of eight peer experts who did not participate in the guideline development process and are responsible for reviewing the guideline recommendations as well as the clarity, feasibility, and endorsement of the guideline.

### 2.3 Target Population and Users of the Guideline

The target population of this guideline is perioperative elderly patients with lung cancer. This guideline is intended for professionals in geriatrics, thoracic surgery, general practice, oncology, anesthesiology, nutrition, and rehabilitation in China.

### 2.4 Selection and Determination of Clinical Question

A systematic search was conducted for existing domestic and international guidelines, systematic reviews, and key original research in areas related to geriatric syndromes, comorbidity status, and complication management in older patients with lung cancer, based on the PICO. In combination with common issues encountered in clinical practice, 29 preliminary clinical questions were proposed. Using a modified Delphi method and scoring by 87 experts and 11 lung cancer patients, 28 clinical questions were ultimately selected.

### 2.5 Evidence Identification, Assessment, and Synthesis

We systematically searched Embase, PubMed, The Cochrane Library, and MEDLINE via OVID, as well as Chinese databases such as CNKI, WanFang Data, and China Biomedical Literature Database (CBM) from inception to August 25, 2025. Additional searches were performed in major guideline repositories, including the National Guideline Clearinghouse (NGC), the Scottish Intercollegiate Guideline Network (SIGN), and the Guidelines International Network (GIN). For each clinical question, two reviewers developed search strategies using controlled vocabulary and free‐text terms. The key search concepts and representative search terms are provided in Table S1.

Eligible evidence included randomized controlled trials (RCTs), cohort studies, case–control studies, systematic reviews, and clinical practice guidelines involving patients aged ≥60 years undergoing lung cancer surgery. Outcomes of interest covered geriatric syndromes, comorbidities, perioperative complications, functional status, symptom burden, rehabilitation, safety, and healthcare utilization. Outcome domains and representative indicators are  shown in Table S2. Study screening, data extraction, and cross‐checking were independently performed by two reviewers, with disagreements resolved by discussion or third‐party arbitration.

Methodological quality and risk of bias were assessed using validated appraisal tools according to evidence type and study design. Details are summarized in Table S3. The GRADE method was used to assess the certainty of evidence and develop recommendations [8]. The certainty of evidence for each outcome was rated by considering five downgrading domains—risk of bias, inconsistency, indirectness, imprecision, and publication bias—and three upgrading domains: large effect size, dose‐response relationship, and possible confounding factors. Good practice statements (GPSs) were used for clinically important recommendations that were not primarily based on direct empirical evidence [9].

### 2.6 Formation of Key Points and Recommendations of the Guideline

On the basis of the grading of evidence levels and the evidence‐to‐decision framework, careful consideration of factors, such as the balance of benefits and harms of interventions, the certainty of evidence, values and preferences, resource use and cost, equity, acceptability, and feasibility, was conducted. Two rounds of Delphi surveys were conducted from October to December 2025 through the “Fangdian: Medical Guideline and Consensus Development Platform” (https://develop.guidelines‐registry.cn/my/guidelines). Through this platform, the working group simultaneously uploaded supporting materials, including recommendations, recommendation rationales, evidence summaries, search strategies, evidence profile tables, and evidence grading tables. The first round of Delphi survey was conducted from October 31 to November 10, 2025, inviting 39 experts to reach consensus, all of whom provided feedback on the recommendations. For the 12 recommendations that failed to reach consensus (consensus rate <80%), a second round of Delphi survey was conducted from December 16 to December 24, 2025, inviting 41 experts to participate; all experts provided feedback. Five recommendations still failed to reach consensus (consensus rate <80%), so final recommendations were not established due to inconsistent expert opinions and insufficient evidence. After the initial draft of the guidelines was completed, eight experts who had not been involved in the earlier drafting process were invited to conduct an external review. After revising based on feedback, the guideline ultimately formed 28 clinical questions and 55 recommendations (Table S4).

## 3 Recommendation and Explanation

### 3.1 Perioperative Management of Geriatric Syndromes in Older Patients With Lung Cancer

#### 3.1.1 Clinical Question 1: How to Conduct Preoperative Frailty Assessment and Management?

##### 3.1.1.1 Recommendation 1

Routine frailty screening is recommended for patients aged ≥65 years undergoing lung cancer surgery (1A). The Geriatric 8 (G8) scale is suggested as the initial screening tool (2A). Patients with a positive G8 screening result (score ≤14) should undergo comprehensive geriatric assessment (CGA) (1B). On the basis of the assessment results, a stratified management approach is recommended: standard enhanced recovery after surgery (ERAS) protocols for non‐frail patients; a “prehabilitation + ERAS” model for prefrail patients; and individualized treatment through multidisciplinary consultation for frail patients (1A).

##### 3.1.1.2 Rationale and Evidence Summary

This recommendation is based on evidence from systematic reviews and meta‐analyses. Frailty is common among older patients with lung cancer and is consistently associated with adverse perioperative and long‐term outcomes, including postoperative complications, prolonged hospitalization, impaired functional recovery, and increased mortality [10, 11, 12, 13, 14, 15, 16, 17, 18, 19, 20, 21, 22]. The working group evaluated 42 studies and found that the prevalence of frailty among older patients with lung cancer was 41% (95% confidence interval [CI], 35–46). Frailty was also associated with poorer overall survival (hazard ratio [HR] = 3.01) and a higher risk of complications (odds ratio [OR] = 1.89) in lung cancer patients [16, 19, 20].

A systematic review of six studies by the working group [10, 11, 12, 13, 14, 15] showed that the G8 scale has high sensitivity for identifying frailty or vulnerability (90%, 95% CI, 87–92) and is easy to administer. A G8 score ≤14 was an independent predictor of mortality risk (HR = 3.21; 95% CI, 2.21–4.66), supporting its use for rapid preoperative identification of high‐risk patients. For patients with a positive G8 screening result (G8 score ≤14), CGA is recommended to inform treatment decisions [23, 24, 25]. CGA further refines risk stratification by predicting mortality, complications, and functional decline and may identify frailty not captured by conventional assessment in approximately 23% of patients [11, 13, 15, 17, 19, 20, 24, 26, 27, 28].

Regarding intervention strategies, the working group synthesized three meta‐analyses (*n* = 5391), which indicated that ERAS tends to reduce postoperative pulmonary complications (PPCs) (risk ratio [RR] = 0.66; 95% CI, 0.59–0.75) and infection rates (RR = 0.52; 95% CI, 0.34–0.80) in lung cancer patients [29, 30, 31]. Additionally, preoperative pulmonary rehabilitation (PR) and multimodal prehabilitation incorporating exercise‐based, nutritional, and psychological interventions improve functional status and reduce PPCs [32, 33, 34]. Therefore, perioperative management should be implemented on the basis of frailty status [35, 36, 37, 38].

#### 3.1.2 Clinical Question 2: How to Assess and Manage Delirium Risk and Implement Effective Interventions?

##### 3.1.2.1 Recommendation 2

Timely identification of potential delirium is recommended for older patients with lung cancer during the perioperative period (1A). Assessment tools, such as confusion assessment method (CAM) or CAM–intensive care unit (ICU), may be used (1B), with an assessment frequency of at least once daily for a minimum of 3 days (2C). Preventive measures should be implemented for high‐risk older patients (Table 1). Delirium prevention should primarily focus on multimodal non‐pharmacological interventions (1B). Routine use of antipsychotic medications for prophylaxis is not recommended (1B). Short‐term, low‐dose, and symptom‐directed use may be considered only when non‐pharmacological measures prove ineffective and significant agitation or safety risks are present (2C).

**TABLE 1:** Risk factor stratification for high‐risk elderly patients.

Risk factors	Classification	Recommendation strength
Advanced age	A	Strong
History of delirium, cognitive impairment/dementia, pain, use of psychotropic medications, preoperative smoking	B	Strong
Weakness	C	Strong
Malnutrition, decreased ADL, impaired IADL, electrolyte imbalance	C	Weak
Preoperative alcohol abuse, concomitant chronic diseases, polypharmacy (≥5 medications)	B	Weak

Abbreviations: ADL, activities of daily living; IADL, instrumental activities of daily living.

##### 3.1.2.2 Rationale and Evidence Summary

This recommendation is supported by high‐quality meta‐analyses. In a meta‐analysis of 12 studies [39, 40, 41, 42, 43, 44, 45], the working group found a pooled postoperative delirium incidence of 16% (95% CI, 12%–20%). A meta‐analysis identified prior delirium, frailty, cognitive impairment, functional decline, psychotropic medication use, and smoking as significant risk factors [46]. These risk factors may occur across the perioperative continuum [47]. Early recognition of delirium is critical for management [48], and systematic review indicates that the CAM is the most used and effective screening tool for postoperative delirium in older patients across various clinical settings [49]. CAM–ICU is routinely recommended for ICU and mechanically ventilated patients. This guideline recommends delirium assessment at least once daily for a minimum duration of 3 days [50]. RCTs showed that haloperidol did not reduce delirium after thoracic surgery [51], and meta‐analyses found no preventive benefit in older postoperative patients [52, 53].

Research indicates that comprehensive non‐pharmacological interventions can reduce delirium incidence by approximately 40% [48]. Effective strategies include precipitating factor control, sleep and pain management, orientation training, and early mobilization [54]. Notably, standardized postoperative analgesia significantly reduces delirium occurrence and severity [55]. Multidisciplinary non‐pharmacological interventions based on Hospital Elder Life Program model represent an effective management framework for reducing perioperative delirium risk [54, 56].

#### 3.1.3 Clinical Question 3: How to Assess the Risk of Perioperative Malnutrition in Older Patients With Lung Cancer?

##### 3.1.3.1 Recommendation 3

Nutrition risk screening 2002 and/or mini‐nutritional assessment (MNA)/mini‐nutritional assessment short form (MNA‐SF) are recommended for nutritional risk screening; the patient‐generated subjective global assessment (PG‐SGA) and/or Global Leadership Initiative on Malnutrition (GLIM) criteria are recommended for the diagnosis and severity grading of malnutrition (2C).

##### 3.1.3.2 Recommendation 4

For patients at risk of dysphagia, swallowing function screening should be initiated as early as possible (2C). The eating assessment tool‐10 (EAT‐10) is recommended as the initial screening tool. For patients with positive screening results or at high risk, the volume–viscosity swallow test (V–VST) is recommended for bedside assessment. Additionally, the Kubota Water Swallow Test, standardized swallowing assessment (SSA), repetitive saliva swallowing test (RSST), or Gugging Swallowing Screen (GUSS) can be used as supplementary tools (GPS). For patients with suspected severe dysphagia or recurrent aspiration, consultation with rehabilitation medicine specialists or swallowing therapists is recommended, with consideration of fiber‐optic endoscopic evaluation of swallowing (FEES) or video fluoroscopic swallowing study (VFSS) (2D) to confirm the diagnosis and guide subsequent clinical interventions (2C).

##### 3.1.3.3 Rationale and Evidence Summary

Nutritional status in older lung cancer patients is an important determinant of postoperative recovery and long‐term prognosis. A meta‐analysis showed that a low prognostic nutritional index was associated with increased mortality in lung cancer patients [57]. Nutrition risk screening 2002 is broadly applicable in older and oncology populations, whereas MNA/MNA‐SF provides a geriatric‐focused assessment of nutritional risk. For diagnosis and grading, PG‐SGA is well validated in cancer populations, and the 2018 GLIM criteria demonstrate acceptable diagnostic performance [58, 59].

Dysphagia is frequent in older adults and contributes to malnutrition and aspiration pneumonia [60, 61, 62]. Because lung cancer‐specific standards are limited, pragmatic use of validated screening and bedside tools is recommended. EAT‐10 exhibits high reliability and validity, making it suitable for rapid initial screening. Meta‐analyses showed that the V–VST has high diagnostic sensitivity and good reliability, representing a preferred bedside assessment tool [63, 64]. VFSS or FEES should be considered when severe dysphagia or recurrent aspiration is suspected. Following diagnosis, the Functional Oral Intake Scale can be used to assess oral intake ability, and the Swallowing Quality of Life can be used to evaluate swallowing‐related quality of life. Integrating functional indicators with patient‐reported outcomes facilitates individualized intervention strategies to reduce complications and improve overall prognosis [65, 66, 67].

#### 3.1.4 Clinical Question 4: How to Formulate Perioperative Nutritional Intervention Strategies?

##### 3.1.4.1 Recommendation 5

Nutritional interventions are recommended based on the principles of “early intervention, individualized management, and multidisciplinary collaboration.” Individualized nutritional goals should be established, with dynamic assessment and adjustment in conjunction with measures such as exercise rehabilitation (GPS). For older patients (≥75 years), those with multiple chronic conditions, or those with frailty, multidisciplinary nutritional management involving or led by the geriatrics department is recommended (1B).

##### 3.1.4.2 Rationale and Evidence Summary

A meta‐analysis of 6 RCTs conducted by the working group [68, 69, 70, 71, 72, 73] revealed that nutritional intervention significantly reduced the risk of postoperative complications (OR = 0.17; 95% CI, 0.10–0.28). Multiple studies confirmed that nutritional intervention can improve postoperative albumin [70, 72, 74, 75, 76], hemoglobin [70, 72, 74], prealbumin, and transferrin levels [69, 72, 74, 75], enhance cellular immune function [70, 72], shorten chest tube duration and hospital stay [68, 69, 73, 74], and improve pulmonary function indices and oxygenation status [71, 77, 78]. Although these findings support the overall effectiveness of perioperative nutritional management, existing studies exhibit significant heterogeneity in intervention forms, implementation timing, and duration, with evidence quality predominantly rated as moderate or low. Therefore, this guideline supports perioperative nutritional intervention as an integral component of comprehensive management but does not make restrictive recommendations regarding specific intervention modalities or periods.

#### 3.1.5 Clinical Question 5: How to Assess and Manage Perioperative Sarcopenia in Older Patients With Lung Cancer?

##### 3.1.5.1 Recommendation 6

Routine preoperative screening and assessment of sarcopenia are recommended (1A). Computed tomography (CT)‐based muscle mass evaluation methods are recommended, particularly using the lumbar 3 (L3) skeletal muscle index (L3MI = (L3 skeletal muscle cross‐sectional area)/(height^2^)) as the diagnostic criterion for sarcopenia, combined with muscle function indicators (such as grip strength, gait speed, or the five‐repetition sit‐to‐stand test) for comprehensive evaluation (2B). For patients diagnosed with sarcopenia, comprehensive management combining exercise intervention and nutritional support is recommended (1A). Preoperative rehabilitation should be incorporated into routine management pathways (1B).

##### 3.1.5.2 Rationale and Evidence Summary

Studies [79, 80, 81] have reported sarcopenia prevalence rates of 42.8%–45% in patients with lung cancer, whereas a systematic review of 30 studies by this guideline working group found a prevalence of 33.2% among older patients with lung cancer. Several studies indicated that sarcopenia is a significant predictor of poor outcomes in older patients with lung cancer, leading to significantly shortened overall survival [79, 82, 83], decreased 5‐year overall survival rates [82], reduced disease‐free survival [84], and significantly increased risk of perioperative complications [80, 84]. Although sarcopenia assessment should ideally include muscle mass, strength, and physical performance, CT‐based muscle measurement is the most feasible and widely supported approach in lung cancer because CT is routinely available for staging and has established prognostic value [82, 84, 85, 86, 87, 88, 89, 90, 91]. Combining CT, preferably L3 skeletal muscle index, with grip strength, gait speed, or the five‐repetition sit‐to‐stand test may further improve diagnostic accuracy. For intervention, the available evidence supports a multimodal strategy centered on exercise and nutritional support. Shen et al.’s meta‐analysis [92] demonstrated that resistance training improves quality of life, grip strength, and gait speed and may be further enhanced by balance or aerobic training. A network meta‐analysis [93] including 59 RCTs and meta‐analysis [94] including 35 RCTs both confirmed that protein supplementation enhances muscle strength and mass. A meta‐analysis comparing different intervention strategies [95] indicated that combined exercise and nutrition interventions are most effective in improving muscle mass. Two meta‐analyses [96, 97] indicated that preoperative exercise training in lung cancer patients can improve pulmonary function, shorten hospital stays, and reduce the risk of postoperative complications. Nevertheless, high‐quality perioperative evidence specific to older patients with lung cancer and sarcopenia remains insufficient.

#### 3.1.6 Clinical Question 6: How to Conduct a Preoperative Fall Risk Assessment?

##### 3.1.6.1 Recommendation 7

Dynamic fall risk assessment is recommended (1A). The Hendrich II fall risk assessment model (HFRM) and Morse Fall Scale (MFS) are recommended as screening tools (1B).

##### 3.1.6.2 Rationale and Evidence Summary

A meta‐analysis of nine studies conducted by the working group demonstrated that history of falls was significantly associated with fall risk in older hospitalized patients and older patients with cancer (RR = 2.66; 95% CI, 1.91–3.71) [98, 99, 100, 101, 102, 103, 104, 105, 106]. The guideline recommends routinely assessing fall history within the previous 12 months to identify high‐risk individuals and guide perioperative comprehensive management [99, 103, 107, 108, 109, 110, 111, 112, 113, 114, 115, 116]. The working group synthesized data from 10 studies on fall risk screening tools [98, 100, 101, 102, 105, 117, 118, 119, 120]. Qualitative analysis consistently supported the combined use of the HFRM and MFS. Furthermore, the HFRM exhibits better sensitivity and specificity compared to the MFS [121], making it more suitable for systematic assessment upon admission or after a fall [106], whereas MFS features a concise structure appropriate for rapid initial screening. The guideline recommends combined application of both tools, with multifactorial, dynamic fall risk assessment conducted for patients identified as high risk at initial screening [105].

#### 3.1.7 Clinical Question 7: How to Implement Early Postoperative Rehabilitation to Prevent Falls?

##### 3.1.7.1 Recommendation 8

CGA (1A) and exercise intervention (1A) are recommended. For patients with fall risk or rehabilitation needs, personalized rehabilitation plans should be developed (2C), and comprehensive rehabilitation management centered on multifactorial intervention should be implemented in the early postoperative period (2B).

##### 3.1.7.2 Rationale and Evidence Summary

Given the limited direct evidence on perioperative fall prevention in older patients with lung cancer, this guideline integrated high‐quality studies from relevant older adults, encompassing seven systematic reviews [122, 123, 124, 125, 126, 127, 128], two meta‐analyses [119, 129], and one RCT [130]. The synthesized evidence indicates that CGA and exercise interventions are key measures for fall prevention. Meta‐analysis confirmed [129] that CGA significantly reduces fall incidence (OR = 0.42). Multiple systematic reviews and RCTs consistently demonstrated [119, 122, 123, 124, 126, 127, 128] that exercise interventions effectively reduced fall risk in older adults with good safety profiles. Therefore, this guideline recommends combining CGA with exercise intervention as a core strategy for fall prevention in older patients.

#### 3.1.8 Clinical Question 8: How to Conduct Pain Assessment and Management?

##### 3.1.8.1 Recommendation 9

Comprehensive pain management throughout the entire perioperative period is recommended. Preoperative pain education is recommended (1C). Intraoperative use of local anesthetics for thoracic paravertebral nerve block, intercostal nerve block, or incisional infiltration aids in preventive analgesia (1B). Postoperatively, recommend daily dynamic assessment of resting and cough‐related pain using standardized scales during hospitalization (2C). Patient‐controlled intravenous analgesia (PCIA) is recommended postoperatively (1C), with scheduled and as‐needed analgesia, primarily using non‐opioid analgesics, supplemented with opioids when necessary (1B).

##### 3.1.8.2 Rationale and Evidence Summary

Studies [131, 132] indicated that preoperative pain education, while not significantly reducing hospital stay duration, improves postoperative recovery metrics, lowers pain scores, and enhances patient satisfaction and analgesic adherence. During hospitalization, daily assessment of resting pain and cough‐induced pain is recommended to dynamically guide analgesic strategy adjustments [133, 134]. Meta‐analyses of intraoperative prophylactic analgesia [135, 136, 137] indicated that different regional analgesic techniques show no significant difference in opioid consumption within 24 h postoperatively, while supporting their role in prophylactic pain management. A systematic review of relevant studies by the working group [138, 139, 140, 141, 142, 143, 144] indicated that postoperative PCIA significantly reduced postoperative pain scores (standard deviation, −2.92 points). Furthermore, based on three RCTs [145, 146, 147], an analgesic strategy primarily based on non‐opioids with on‐demand opioid supplementation showed a favorable trend in reducing the incidence of postoperative nausea and vomiting within 0–24 h postoperatively (RR = 0.68). Implementing this strategy tailored to individual patient risk is recommended.

#### 3.1.9 Clinical Question 9: How to Intervene in Postoperative Sleep Disturbance?

##### 3.1.9.1 Recommendation 10

It is recommended to conduct baseline screening using the Pittsburgh sleep quality index (PSQI) preoperatively and dynamic assessment using Richards‐Campbell Sleep Questionnaire (RCSQ) or numerical rating scale (NRS) postoperatively. Intervention for postoperative sleep disturbance should be initiated when RCSQ <50 or NRS <5 (1B).

##### 3.1.9.2 Recommendation 11

For patients with a preoperative PSQI ≥5 and postoperative RCSQ <50 or NRS <5, targeted interventions should be implemented on the basis of the main influencing factors, including perioperative cognitive behavioral therapy (CBT) delivered by multidisciplinary treatment (MDT) and multimodal analgesia management centered on combined stellate ganglion block (SGB) and PCIA during surgery (1B).

##### 3.1.9.3 Rationale and Evidence Summary

Research indicates that perioperative sleep disorders occur in over 70% of patients undergoing lung cancer surgery [148]. They are closely associated with pain, coughing, and psychological stress [148, 149, 150], often significantly impacting postoperative recovery. PSQI demonstrates good reliability and validity in cancer patients and serves as a reliable tool for identifying pre‐existing sleep problems, with a PSQI score ≥5 defining high risk for sleep disturbance. However, its retrospective recall period is relatively long, making it unsuitable for repeated acute‐phase assessments [151, 152, 153]. In contrast, RCSQ demonstrated good reliability and validity in both ICU and general ward settings, while NRS for sleep quality is simple to administer, making both more suitable for continuous dynamic assessment during hospitalization [154].

Interventions for postoperative sleep disorders in older patients with lung cancer primarily include non‐pharmacological interventions, multimodal pain management, pharmacological interventions, and integrated traditional Chinese medicine protocols. Evidence supports implementation of nursing interventions on the basis of CBT during the perioperative period, combined with multimodal analgesia management, including intraoperative SGB and postoperative PCIA, which can effectively relieve pain, improve sleep architecture, and shorten hospitalization duration [155, 156, 157, 158]. Currently, there is insufficient evidence to support the routine use of pharmacological interventions or integrated traditional Chinese medicine approaches.

#### 3.1.10 Clinical Question 10: How to Assess Activities of Daily Living (ADL)/Instrumental Activities of Daily Living (IADL) and Set Postoperative Rehabilitation Goals?

##### 3.1.10.1 Recommendation 12

It is recommended to assess ADL/IADL using the Katz‐15 scale (2D). Postoperative rehabilitation goals should be established following the principle of being “achievable, quantifiable, evaluable, and time‐bound.” Patients should be encouraged to actively participate in goal setting (GPS).

##### 3.1.10.2 Rationale and Evidence Summary

Although the existing evidence is of low quality, this intervention exhibits favorable safety profiles, low costs, and the expert panel unanimously agreed on its potential positive significance for functional recovery in patients. The incidence of preoperative ADL/IADL dependence is relatively high in older patients with lung cancer [159, 160]. The combination of Katz‐6 and Lawton IADL forms the Katz‐15 scale [161], which demonstrates good internal consistency and superior predictive performance compared to Katz‐6 alone [162]. Multiple systematic reviews and meta‐analyses [163, 164, 165, 166] have shown that participation in goal setting improves quality of life and emotional status, whereas structured goal setting further enhances self‐efficacy and satisfaction. Existing research recommends promoting patient engagement throughout the process [167, 168]. Further high‐quality research is needed to validate the implementation details and cost‐effectiveness of this approach.

#### 3.1.11 Clinical Question 11: How to Assess and Intervene for Anxiety and Depression?

##### 3.1.11.1 Recommendation 13

It is recommended to prioritize the use of the geriatric depression scale (GDS)‐15 (or GDS‐11 for Chinese‐speaking patients) for depression assessment (1B). The geriatric anxiety inventory (GAI) is recommended for anxiety screening, with threshold adjustment appropriate to the characteristics of the lung cancer population (2B).

##### 3.1.11.2 Recommendation 14

For patients aged ≥65 years undergoing thoracoscopic radical resection for lung cancer, after excluding contraindications and conducting individualized assessment, consider perioperative low‐dose esketamine administration to reduce postoperative depression and anxiety scores. Mental status should be closely monitored during use, with vigilance for drug‐related adverse reactions (2B).

##### 3.1.11.3 Recommendation 15

Non‐pharmacological interventions are recommended as the foundational strategy. CBT is effective in alleviating anxiety symptoms (1B) and shows a potential favorable trend in improving depression scores (2C). Additionally, a multidisciplinary integrated care model is recommended (1B) to promote comprehensive patient recovery.

##### 3.1.11.4 Rationale and Evidence Summary

For depression screening, the GDS‐15 demonstrates good reliability and validity among older adults in China, whereas the GDS‐11 version is better structured to align with the characteristics of older adults in both urban and rural Chinese settings [169, 170]. For anxiety screening, the GAI shows reliable validity in older populations [171, 172]. However, for patients with chronic respiratory diseases such as lung cancer, it is proposed to adopt lower modified cut‐off values in conjunction with clinical interviews for comprehensive assessment. A meta‐analysis of six RCTs included by this guideline working group [173, 174, 175, 176, 177, 178] demonstrated that perioperative esketamine administration significantly reduced postoperative depression incidence and effectively alleviated postoperative depression and anxiety scores. Regarding non‐pharmacological interventions, systematic reviews and meta‐analyses indicated that CBT significantly reduces perioperative anxiety scores and shows consistent potential benefits for depressive outcomes [179, 180, 181, 182, 183, 184, 185]. A meta‐analysis of 3 RCTs conducted by the guideline working group demonstrated [78, 186, 187] that multidisciplinary integrated care models significantly reduce perioperative anxiety and depression in patients.

### 3.2 Perioperative Comorbidity Management in Older Patients With Lung Cancer

#### 3.2.1 Clinical Question 12: How to Assess the Risk of Major Perioperative Cardiovascular Events?

##### 3.2.1.1 Recommendation 16

Systematic preoperative assessment of medical history and cardiovascular risk factors is recommended, combined with clinical risk scoring tools and functional status evaluation to complete preliminary risk stratification (2B).

##### 3.2.1.2 Recommendation 17

For patients aged ≥65 years or with prior history of chronic cardiovascular disease, comprehensive perioperative cardiovascular risk assessment is required, with dynamic monitoring of cardiac biomarkers and electrocardiogram within 72 h postoperatively (1B).

##### 3.2.1.3 Recommendation 18

For patients presenting with new‐onset dyspnea, suspected heart failure, or high‐risk factors including significant valvular disease, arrhythmia, or pulmonary hypertension, as well as those aged ≥75 years, transthoracic echocardiography and coronary CT angiography should be considered to clarify cardiac function and structural abnormalities. Specific protocols should be determined through shared decision‐making between MDT and patient to establish optimal surgical timing (1B).

##### 3.2.1.4 Recommendation 19

For patients with recent or worsening unstable acute coronary syndrome, hemodynamic instability, uncontrolled heart failure, or severe ventricular arrhythmias, elective surgery should be postponed in favor of prioritized cardiovascular optimization, with MDT management when indicated (1B).

##### 3.2.1.5 Rationale and Evidence Summary

Existing evidence supports the use of the revised cardiac risk index (RCRI) [188, 189] or the American College of Surgeons National Surgical Quality Improvement Program (ACS‐NSQIP) risk calculator [188, 189, 190], together with functional status evaluation, for postoperative cardiovascular risk stratification [191, 192, 193, 194]. Exercise capacity <4 METs is associated with higher postoperative major adverse cardiovascular events (MACE), prolonged hospitalization, and mortality; conversely, asymptomatic patients with preserved functional capacity usually do not require further cardiovascular testing before elective noncardiac surgery [195, 196].

B‐type natriuretic peptide (BNP), N‐terminal pro‐B‐type natriuretic peptide (NT‐proBNP), and troponin provide feasible markers of ventricular stress and myocardial injury [197, 198], and preoperative BNP or NT‐proBNP predicts major postoperative cardiovascular complications [197, 199, 200, 201, 202, 203, 204, 205]. Routine echocardiography or coronary CT angiography is not recommended for asymptomatic individuals without clinical indications [206, 207, 208]. For high‐risk patients, echocardiography and/or coronary CT angiography may refine risk assessment, clarify cardiac structure or coronary disease, and inform perioperative planning [209, 210, 211, 212, 213]. Surgery should be deferred in unstable acute coronary syndrome, hemodynamic instability, uncontrolled heart failure, or severe ventricular arrhythmias until cardiovascular status is optimized, preferably through multidisciplinary evaluation [206, 207, 208].

#### 3.2.2 Clinical Question 13: How to Optimize Perioperative Medication Management in Patients With Hypertension, Coronary Artery Disease, Chronic Heart Failure, and Atrial Fibrillation?

##### 3.2.2.1 Recommendation 20

Patients on long‐term β‐blockers (1B), calcium channel blockers (CCBs) (2C), or diuretics (2C) for blood pressure control should continue these medications throughout the perioperative period, with close monitoring of heart rate, blood pressure, electrolytes, and electrocardiographic activity. Angiotensin receptor–neprilysin inhibitors, angiotensin‐converting enzyme inhibitors (ACEIs), and angiotensin receptor blockers (ARBs) should be withheld 24 h prior to surgery. In hemodynamically stable patients, these medications can be resumed on the second postoperative day (1B). The use of α‐blockers for perioperative blood pressure control is not recommended (2C).

##### 3.2.2.2 Recommendation 21

Patients with coronary heart disease receiving single antiplatelet therapy for primary prevention should discontinue the medication. Whether to discontinue for secondary prevention requires evaluation by an MDT. Aspirin should be discontinued for ≥7 days, clopidogrel for ≥5 days, and ticagrelor for ≥3 days before surgery (1B). Surgery is not recommended for patients on dual antiplatelet therapy (DAPT) (1A). If surgery is necessary, it is advisable to postpone the surgery until at least 6 weeks after percutaneous coronary intervention (PCI) and discontinue one of the antiplatelet agents (2C).

##### 3.2.2.3 Recommendation 22

For patients receiving novel oral anticoagulants (NOACs), discontinuation 48 h preoperatively is recommended, with resumption 48–72 h postoperatively (1B). For patients with atrial fibrillation on warfarin, it should be discontinued at least 5 days before surgery, with management stratified based on congestive heart failure, hypertension, age ≥75 years (2 points), diabetes mellitus, stroke or transient ischemic attack (2 points), vascular disease, age 65–74 years, sex category (CHA_2_DS_2_‐VASc) score. Heparin bridging is not recommended for patients without a high thromboembolic risk. The preoperative international normalized ratio (INR) should be maintained below 1.5, and anticoagulation should be resumed 48–72 h postoperatively (1B). For patients at a high thromboembolic risk, heparin bridging may be considered, with anticoagulation resuming 48–72 h postoperatively. The decision regarding bridging therapy should arise from an MDT discussion, enhancing the prevention of the perioperative bleeding risks (2C).

##### 3.2.2.4 Recommendation 23

For older lung cancer patients with cardiac dysfunction, surgical decision and perioperative management plans should be collaboratively developed through MDT discussion (2C).

##### 3.2.2.5 Rationale and Evidence Summary

Angiotensin receptor–neprilysin inhibitors (ARNI)/ACEI/ARB therapy may increase intraoperative hypotension [214, 215, 216]. Given the association between intraoperative hypotension and poorer overall survival [217], blood pressure should be closely monitored and ARNI/ACEI/ARB therapy withheld 24 h before surgery. β‐blockers may reduce perioperative mortality, myocardial infarction (MI), and atrial fibrillation but increase bradycardia and hypotension [218, 219, 220, 221, 222]. CCBs can generally be continued [223]. Diuretic management should be individualized, as diuretic observational data suggest risks of hypotension and kidney injury, whereas RCT evidence remains inconclusive [224, 225, 226, 227, 228, 229]. α‐blockers are not recommended because of increased risks of hypotension, cardiac arrest, and falls [230, 231, 232].

Antiplatelet therapy should be managed according to thrombotic risk, bleeding risk, and multidisciplinary assessment. Temporary interruption of single antiplatelet therapy does not appear to increase mortality, bleeding, or ischemic events [233, 234, 235, 236, 237, 238, 239, 240], and discontinuation timing should be based on drug half‐life and bleeding risk [241, 242, 243]. Elective surgery should be avoided during mandatory DAPT, particularly within 6 weeks after PCI, because of the high risk of stent thrombosis, MI, and mortality [244]. In urgent surgery, tirofiban bridging therapy may be considered, but its safety within the first month after PCI is uncertain [245].

For patients on NOACs, temporary perioperative interruption is associated with low risks of thromboembolism, major bleeding, and all‐cause mortality [246]. In atrial fibrillation patients on warfarin without high thromboembolic risk, routine heparin bridging is not recommended because it increases major bleeding without reducing thrombotic events [247, 248, 249]. In high‐thrombotic‐risk patients, including those with a CHA_2_DS_2_‐VASc score ≥5, bridging may still be considered under close monitoring, although supporting evidence is limited [208, 241, 242].

#### 3.2.3 Clinical Question 14: How to Assess the Perioperative Risk in Patients With Chronic Obstructive Pulmonary Disease (COPD)?

##### 3.2.3.1 Recommendation 24

COPD is an important risk factor for surgery in older patients with lung cancer, particularly those aged ≥75 years (1B). Predicted postoperative forced expiratory volume in one second (ppo‐FEV_1_) and predicted postoperative transfer factor for carbon monoxide (ppo‐TLCO) serve as crucial parameters for evaluating perioperative risk (1B). Cardiopulmonary exercise testing (CPET) is recommended, and if the decline in peripheral oxygen saturation (SpO_2_) exceeds 15%, surgical indications should be carefully evaluated (1B). Quantitative CT can aid in assessing perioperative risk in patients with COPD (2B).

##### 3.2.3.2 Rationale and Evidence Summary

COPD is an independent risk factor for postoperative cardiopulmonary complications. Studies have shown that the risk of postoperative complications increases in patients older than 75 years, with age carrying greater prognostic weight as the stage of lung cancer advances [250]. FEV_1_ and TLCO are critical parameters for perioperative risk assessment in patients with COPD [251, 252, 253, 254, 255]. Bronchodilators can improve forced expiratory volume in one second (FEV_1_) and dyspnea symptoms but do not affect overall survival [253, 256, 257]. FEV_1_ and TLCO <60% of predicted indicate high surgical risk, necessitating CPET. Among individuals undergoing lobectomy with FEV_1_ and TLCO <60% of the predicted value, those who experience a decline in SpO_2_ exceeding 4% are associated with a significant increase in cardiopulmonary complications and mortality [258]. If a patient exhibits a decline in SpO_2_ greater than 15%, careful consideration of surgical intervention is warranted [259]. Quantitative CT possesses predictive value for postoperative complications and survival; however, most of these studies have been retrospective studies [260, 261, 262]. Generally, patients with ppo‐FEV_1_ and ppo‐TLCO ≥80% of the predicted values and no other risk factors have a low surgical risk. Patients with <40% predicted value demonstrate elevated 1‐month postoperative mortality, though long‐term survival remains superior to nonsurgical patients [263, 264].

#### 3.2.4 Clinical Question 15: How to Develop Preoperative and Postoperative PR Strategies for Patients With COPD?

##### 3.2.4.1 Recommendation 25

Perioperative PR is recommended for older patients with lung cancer and comorbid COPD. PR should be initiated 2–4 weeks prior to surgery. The preoperative PR program should include respiratory function training, high‐intensity exercise training, nutritional management, and psychological education. Weekly monitoring of pulmonary function, exercise tolerance, and other relevant indicators is essential to guide program adjustments. Postoperative rehabilitation should begin as early as possible, with early rehabilitation initiated on the first postoperative day. This structured approach should progress sequentially through the acute phase, recovery phase, discharge transition phase, and long‐term maintenance phase (1A).

##### 3.2.4.2 Rationale and Evidence Summary

Patients with lung cancer and comorbid COPD are at significantly increased risk for surgical complications and postoperative adverse events due to decreased pulmonary function and exercise tolerance [265, 266, 267, 268]. PR has been shown to reduce dyspnea, enhance quality of life, and shorten the length of hospital stay [269, 270, 271]. Numerous studies have confirmed that perioperative PR offers significant benefits to older patients with lung cancer and COPD [272, 273, 274, 275]. A meta‐analysis suggested that PR reduces the risk of postoperative complications (OR = 0.21; 95% CI, 0.12–0.37) and the incidence of pneumonia (OR = 0.36; 95% CI, 0.15–0.86) [276]. Findings from an RCT involving 74 participants revealed significant improvements in both peak oxygen consumption and 6‐min walk test distance in the exercise group [275]. Furthermore, preoperative systematic rehabilitation can reduce postoperative hospital stay and shorten the duration of antibiotic use [274, 276].

#### 3.2.5 Clinical Question 16: How to Establish Glycemic Control Targets and Management Protocols for Patients With Comorbid Diabetes?

##### 3.2.5.1 Recommendation 26

A comprehensive evaluation of the patient's diabetes history should be conducted, including the measurement of fasting blood glucose (FBG) and hemoglobin A1c (HbA1c) to assess glycemic control status and identify any diabetic complications before surgery. Continuous glycemic monitoring during surgery is recommended to guide adjustments in insulin dosage and administration. Postoperative FBG should be maintained below 10 mmol/L with dynamic monitoring to reduce the risk of infection (2C).

##### 3.2.5.2 Rationale and Evidence Summary

The presence of diabetes or hyperglycemia in older patients with lung cancer is associated with an increased incidence of perioperative complications, extended hospital stays, and greater healthcare burden [277, 278, 279, 280]. Multiple studies and RCTs have demonstrated that standardized perioperative glycemic management can reduce postoperative complications and mortality, shorten the duration of hospital stays and mechanical ventilation, and improve long‐term outcomes for patients undergoing lung cancer surgery [281, 282, 283, 284, 285, 286, 287]. Evidence from RCTs supports preoperative blood glucose control at 4.4–6.1 mmol/L of FBG and postoperative 6.1–9.0 mmol/L, or FBG <10 mmol/L, 2‐h postprandial blood glucose <12 mmol/L, which significantly reduces complications, inflammatory responses, and mortality [285, 286, 287]. Given the increased risk of perioperative ketoacidosis associated with sodium‐dependent glucose transporter 2 inhibitor (SGLT2i), these agents should be discontinued preoperatively and replaced with insulin regimens for glycemic control [14].

#### 3.2.6 Clinical Question 17: How to Manage Medications in Patients With Comorbid Chronic Kidney Disease (CKD)?

##### 3.2.6.1 Recommendation 27

For older patients with lung cancer and comorbid CKD and cardiovascular diseases (CVD), ACEIs are preferred for blood pressure control, whereas ARBs may serve as an alternative. The combination of ACEIs and ARBs should be avoided (1B).

##### 3.2.6.2 Recommendation 28

Lipid management is indicated for all older patients with lung cancer and comorbid CKD and CVD, with statins being the first‐line therapy. If low‐density lipoprotein cholesterol targets are not met or if statins are not tolerated, cholesterol absorption inhibitors or proprotein convertase subtilisin/kexin type 9 (PCSK9) inhibitors may be used either in combination or as alternatives (1B).

##### 3.2.6.3 Recommendation 29

For older lung cancer patients with comorbid Type 2 diabetes mellitus (T2DM) and CKD, SGLT2is are recommended, although they should be discontinued on the day of surgery. If glycemic targets are not achieved or SGLT2is are not tolerated, glucagon‐like peptide‐1 (GLP‐1) receptor agonists may be considered. The dosage of metformin should be adjusted based on renal function: contraindicated when the estimated glomerular filtration rate (eGFR) <30 mL/min/1.73 m^2^; not recommended as initial therapy when the eGFR <45 mL/min/1.73 m^2^, and dose reduction is necessary for patients already receiving it (1B).

##### 3.2.6.4 Rationale and Evidence Summary

Evidence from network meta‐analysis and large‐scale RCT synthesis suggests that ACEIs may be more effective than ARBs in reducing kidney failure, cardiovascular mortality, and all‐cause mortality [288, 289]. ACEI–ARB combination therapy should be avoided because of increased hyperkalemia risk [288]. Renal function and serum potassium should be monitored after treatment initiation or escalation, and therapy should be withheld if eGFR decreases by >30% within 3 months.

A meta‐analysis demonstrated that rosuvastatin and atorvastatin significantly reduce the risk of MACE in non‐dialysis CKD patients [290]. When monotherapy proves insufficient, combination therapy with ezetimibe [290, 291] or PCSK9 inhibitors [292] may offer additional benefits, supported by RCT evidence [293]. Evidence for newer agents remains limited in advanced CKD and dialysis patients [292].

Glucose‐lowering therapy should be individualized according to renal function, cardiovascular benefit, and perioperative risk [294]. SGLT2is and GLP‐1 receptor agonists reduce adverse renal outcomes and mortality in selected patients [295, 296, 297, 298, 299, 300]. SGLT2is should be withheld on the day of surgery to reduce the risk of postoperative ketoacidosis [301]. Metformin remains the first‐line therapy for T2DM, but renal function must be carefully assessed: Initiation is not recommended or dose reduction is required when eGFR <45 mL/min/1.73 m^2^, and use is contraindicated when eGFR <30 mL/min/1.73 m^2^ [302, 303].

#### 3.2.7 Clinical Question 18: How to Assess, Monitor, and Correct Patients With Anemia?

##### 3.2.7.1 Recommendation 30

It is recommended to complete universal screening for anemia and etiological classification as early as possible preoperatively. Early identification and targeted intervention can reduce perioperative risks and improve clinical outcomes (1A).

##### 3.2.7.2 Recommendation 31

For patients with iron deficiency anemia, intravenous iron is the preferred treatment. Oral iron may also be considered if the preoperative window allows sufficient time. For non‐iron deficiency anemia, management should be individualized based on the specific underlying etiology. Perioperative blood transfusions should follow a restrictive threshold strategy (1B).

##### 3.2.7.3 Recommendation 32

For patients with significant perioperative blood loss, limited time for correction, or inadequate response to iron therapy, an MDT should evaluate the initiation of erythropoiesis‐stimulating agents (ESAs). After excluding contraindications, short‐term ESAs may be administered in conjunction with iron supplementation, with dynamic assessment of thromboembolic risk and individualized treatment under close monitoring (2B).

##### 3.2.7.4 Recommendation 33

Throughout the perioperative period, hemoglobin and iron metabolism parameters should be dynamically monitored, with reassessments occurring on postoperative Day 3 and prior to discharge. It is recommended to integrate anemia management into the thoracic surgery ERAS pathway and MDT framework to standardize implementation and ensure systematic follow‐up (1B).

##### 3.2.7.5 Rationale and Evidence Summary

A meta‐analysis reported that the risk of mortality in patients with preoperative anemia is increased 1.6‐fold (HR = 1.58; 95% CI, 1.44–1.75) [304]. High‐quality RCTs and international consensus have established that preoperative intravenous iron supplementation significantly increases hemoglobin levels in a short timeframe, reduces the need for allogeneic transfusions, and demonstrates a favorable safety profile [305, 306]. Furthermore, for patients experiencing anemia of chronic disease or insufficient response to single iron therapy, short‐term perioperative combination therapy involving ESAs and iron has been shown to enhance hemoglobin recovery and improve the quality of postoperative recovery [306].

The core principles of this recommendation include early identification, targeted intervention, restrictive transfusion, and multidisciplinary collaboration. A comprehensive assessment of laboratory indicators, including hemoglobin, ferritin, transferrin saturation, vitamin B12, and folate, should be performed preoperatively to establish the type of anemia. For patients diagnosed with iron deficiency anemia, intravenous iron is preferred to minimize intraoperative transfusion needs [306, 307]. In hemodynamically stable patients, strict adherence to restrictive transfusion thresholds is necessary to reduce the risks associated with transfusion [308, 309].

#### 3.2.8 Clinical Question 19: How to Monitor Liver Function and Manage Medication for Patients With Liver Dysfunction?

##### 3.2.8.1 Recommendation 34

For older patients with lung cancer and concomitant hepatic impairment, liver function should be assessed preoperatively, and INR and liver reserve should be measured simultaneously. Patients should be classified using the Child‐Pugh or model for end‐stage liver disease (MELD) score, and perioperative medication should be adjusted accordingly (2C).

##### 3.2.8.2 Rationale and Evidence Summary

The Child‐Pugh score is an effective tool for assessing preoperative liver function [310]. Patients with hepatic impairment should be evaluated for coagulation function, and any abnormalities should be corrected promptly; thromboelastographic and thrombin generation assays should be performed when available [311]. Surgical contraindications include: acute hepatitis or active alcoholic hepatitis; Child‐Pugh Class C; MELD score >15; or significant extrahepatic organ dysfunction [311]. Avoid using drugs that cause liver damage and using antibiotics to prevent postoperative infections [312]. One RCT found [313] that patients with concomitant hepatic impairment may be a potential beneficiary group for the combination of the new benzodiazepine remimazolam and general anesthesia. In older patients with lung cancer and hepatic impairment, reduced hepatic reserve, impaired drug clearance, and decreased synthesis of coagulation factors increase the risk of perioperative complications such as drug accumulation, liver failure, and bleeding. The relevant evidence primarily comes from pharmacokinetic studies and expert consensus [314, 315].

#### 3.2.9 Clinical Question 20: How to Assess and Manage the Risk of Stroke?

##### 3.2.9.1 Recommendation 35

It is recommended to prevent venous thrombosis in older patients, physically inactive patients, patients with a heavy tumor burden, and those undergoing thoracotomy for lung cancer during the perioperative period. Routine monitoring of serum D‐dimer levels should be conducted. If the level is significantly elevated, the risk of thrombosis and stroke is evaluated. After ruling out contraindications for bleeding, prophylactic anticoagulation should be considered (2C).

##### 3.2.9.2 Recommendation 36

The frequency of brain magnetic resonance imaging (MRI) screening is determined based on tumor staging, molecular subtyping, and disease progression. For patients at high risk of brain metastasis, the evaluation frequency should be increased for early detection of brain metastasis or cerebrovascular events (2C).

##### 3.2.9.3 Recommendation 37

For acute ischemic stroke with appropriate indications and no contraindications, mechanical thrombectomy for reperfusion therapy is preferred. This is particularly suitable for patients with unstable surgical wounds, active tumors, or those requiring continued anticoagulation, as it can safely and rapidly restore blood flow and preserve neurological function (2D).

##### 3.2.9.4 Recommendation 38

Pay attention to the blood uric acid level. For patients with persistently low levels (<4 mg/dL), optimize their metabolic and nutritional status (2D).

##### 3.2.9.5 Rationale and Evidence Summary

A systematic review of 11 studies by the working group [316, 317, 318, 319, 320, 321, 322, 323, 324, 325, 326] showed that elevated D‐dimer levels significantly increase the risk of cerebral infarction (RR = 1.92; 95% CI, 1.04–3.56) [317, 319, 326]; elevated platelet counts are also a risk factor for ischemic stroke (RR = 2.73; 95% CI, 1.81–4.11), whereas low‐molecular‐weight heparin therapy reduces the risk of thrombosis by 34%, and the risk of recurrence decreases by 12% for every additional month of treatment [370, 371, 372]. Uric acid levels <4 mg/dL are negatively associated with acute ischemic stroke; for every 1 mg/dL decrease, the risk increases by 1.5% [316], although the study sample size was limited. Regarding clinical assessment, patients with a Khorana score ≥3 had a 73% increased risk of stroke (HR = 1.73; 95% CI, 1.32–2.25) and a 66% increased risk of all‐cause mortality (HR = 1.66; 95% CI, 1.48–1.87), with the best predictive performance observed in patients receiving radiotherapy [325]. Imaging studies show a significantly elevated incidence of cerebral metastases in patients with cerebral infarction, with a 5.2‐fold increased risk in those with multiple infarcts [320]. A National Institutes of Health Stroke Scale neurological score >10 is an independent predictor of poor prognosis; for every 1‐point increase in the score, the 90‐day post‐discharge mortality rate rises by 13.6% [318, 324]. Intervention studies have shown [322, 323] that mechanical thrombectomy significantly improves neurological function compared to intravenous thrombolysis and achieves a higher rate of successful reperfusion in patients with active cancer.

#### 3.2.10 Clinical Question 21: How to Manage Perioperative Polypharmacy?

##### 3.2.10.1 Recommendation 39

Periodic medication reviews are recommended (1B) to reduce inappropriate medication use and improve medication adherence through interventions such as prescription simplification (1B), patient education (1C), and medication nursing (1C).

##### 3.2.10.2 Rationale and Evidence Summary

A meta‐analysis [327] showed that medication reviews can reduce readmission rates and all‐cause mortality. High‐quality RCTs have also confirmed [328] that multimodal interventions can reduce emergency department visits and 30‐day readmission rates. A meta‐analysis of 32 RCTs indicated [329] that medication simplification significantly reduces potential inappropriate medication use, potential prescribing omissions (PPOs), and adverse drug reactions in older adults, while improving medication adherence. Behavioral interventions or education combined with behavioral interventions can also improve long‐term medication adherence in patients [330]. A Cochrane systematic review suggests that interventions, such as medication management, have limited effects on reducing PPOs and carry a risk of bias [331]. Perioperative management of chronic diseases should involve individualized decision‐making based on key factors such as the risk of medication rebound or disease progression, changes in drug absorption, the risk of anesthesia and surgical complications, the necessity of short‐term treatment, and drug interactions [332].

### 3.3 Management of Perioperative Complications in Older Patients With Lung Cancer

#### 3.3.1 Clinical Question 22: How to Prevent and Early Identify PPCs?

##### 3.3.1.1 Recommendation 40

It is recommended to implement CGA combined with pulmonary function stratification. Use the Assess Respiratory Risk in Surgical Patients in Catalonia (ARISCAT) score to assess the risk of PPCs and intervene based on the risk level. For high‐risk patients, multimodal pre‐rehabilitation should be initiated 4–6 weeks before surgery (1A).

##### 3.3.1.2 Recommendation 41

Use protective lung ventilation and restrictive/targeted fluid management, continuously monitor respiratory mechanics, and reduce ventilation‐associated lung injury and acute respiratory distress syndrome (1B).

##### 3.3.1.3 Recommendation 42

The I COUGH strategy is recommended for postoperative PR, using multimodal or regional analgesia to ensure deep breathing and effective coughing, combined with nutritional and electrolyte optimization, to reduce the risk of atelectasis, infection, and hypoxia (2A).

##### 3.3.1.4 Recommendation 43

Conduct intensive monitoring 48–72 h after surgery. Use standardized criteria, such as the European Perioperative Clinical Outcome or the Melbourne Group Scale, to determine PPCs. Establish a continuous care pathway and audit metrics through collaboration among thoracic surgery, anesthesiology, respiratory/rehabilitation, geriatrics, and nutrition teams (1B).

##### 3.3.1.5 Rationale and Evidence Summary

A systematic review [333] showed that the ARISCAT score had the best discriminatory power and recommended its routine use for risk stratification in older patients with lung cancer. A meta‐analysis [334] indicated that smoking within 4 weeks prior to surgery was associated with a higher incidence of postoperative complications. Another systematic review [335] showed that patients who had quit smoking for more than 4 and 8 weeks prior to surgery had a lower risk of respiratory complications compared to current smokers and recommended quitting smoking for at least 4–8 weeks before surgery. Regarding intraoperative airway management, a systematic review [336] showed that the use of low tidal volumes combined with positive end‐expiratory pressure significantly reduced the incidence of postoperative pulmonary infections, atelectasis, acute lung injury, and length of hospital stay. Large‐scale clinical studies have shown [337] that implementation of the I COUGH strategy reduced the incidence of postoperative pneumonia from 2.6% to 1.6% and the rate of unplanned intubation from 2.0% to 1.2%. A systematic review synthesized the effects of measures such as the ERAS pathway, prophylactic mucolytics, prophylactic respiratory physiotherapy, and goal‐directed hemodynamic management on PPCs [338, 339]. Although some studies suggest potential benefits, these measures were not included in the core recommendations due to high heterogeneity and moderate‐to‐low quality of evidence; however, they hold exploratory value.

#### 3.3.2 Clinical Question 23: What Are the Procedures for the Early Identification and Management of MI/Heart Failure?

##### 3.3.2.1 Recommendation 44

Comprehensive cardiac assessment is recommended. Cardiac biomarkers should be monitored preoperatively and within 48–72 h postoperatively. Cardiovascular risk stratification should be performed, and cardiac imaging should be selected individually to enable early identification of MI and heart failure (1B).

##### 3.3.2.2 Recommendation 45

Individualized selection of interventional or surgical treatment versus conservative pharmacological management is recommended for older patients based on the type of perioperative MI and the risk associated with coronary revascularization (1B). In patients with chronic heart failure, continuation of the pre‐existing treatment regimen is recommended; in those with acute heart failure, immediate pharmacological and non‐pharmacological interventions should be initiated (1C). An MDT collaborative model is recommended (1B).

##### 3.3.2.3 Rationale and Evidence Summary

Cohort studies and meta‐analyses have demonstrated that elevated levels of hs‐Tn/cTn and BNP/NT‐proBNP are significantly associated with an increased risk of postoperative MACE [340, 341, 342, 343, 344, 345]. Perioperative MI is often insidious but carries a serious prognosis; therefore, the benefits of active monitoring clearly outweigh the costs and risks. A prospective cohort study in the United Kingdom (*n* = 478,000) showed that the cumulative incidence of CVD during follow‐up in lung cancer patients can reach 22.8% [346]. Commonly used CVD risk assessment tools include the RCRI [347], Thoracic Risk Calculation Index [348], ACS‐NSQIP surgical risk calculator [349], and the novel AUB‐HAS2 cardiovascular risk index [350, 351]. Electrocardiogram, echocardiography, coronary CT angiography, and CT‐based coronary artery calcium score are also important adjunctive tools for cardiac risk assessment [213, 352, 353, 354, 355, 356, 357, 358].

Existing studies and meta‐analyses indicate that emergency PCI is the preferred reperfusion strategy for ST‐elevation MI (STEMI), whereas an invasive strategy may be selected for non‐STEMI [359, 360, 361, 362, 363, 364]. MDT management optimizes decision‐making, improves outcomes, and reduces healthcare resource utilization [365, 366, 367, 368, 369, 370, 371], which is consistent with international guideline recommendations [208, 372].

#### 3.3.3 Clinical Question 24: Caprini Score for Deep Vein Thrombosis and Timing of Anticoagulation

##### 3.3.3.1 Recommendation 46

The Caprini risk assessment model is recommended for evaluating the risk of venous thromboembolism (VTE) (1B), and stratified prevention strategies should be implemented on the basis of the risk score. For high‐risk patients (score ≥5), pharmacological prophylaxis combined with mechanical prophylaxis is recommended (2C).

##### 3.3.3.2 Recommendation 47

Anticoagulant medications should be discontinued 12–24 h prior to surgery. Postoperatively, anticoagulation therapy should be resumed within 12–24 h based on bleeding risk assessment, and extended prophylaxis up to 4 weeks post‐surgery should be considered (1B). Low molecular weight heparin is the preferred pharmacological agent for VTE prophylaxis (2C).

##### 3.3.3.3 Rationale and Evidence Summary

Recommendation 46 is based on 8 cohort studies [373, 374, 375, 376, 377, 378, 379, 380]. The Caprini score demonstrates good predictive value for perioperative VTE risk in older patients with lung cancer. Due to heterogeneity in Caprini score stratification criteria, VTE definitions, and follow‐up durations across studies, a qualitative analysis was conducted. A total of six studies [381, 382, 383, 384, 385, 386] were included in this guideline to evaluate stratified anticoagulation prophylaxis strategies, including three meta‐analyses/systematic reviews and three cohort studies. Stratified prophylaxis strategies may reduce the incidence of VTE.

A prospective cohort study provided evidence supporting the timing of early postoperative anticoagulation resumption [377]. Hong et al. [387] investigated the safety of continuing perioperative antiplatelet therapy in older patients with lung cancer aged ≥60 years, which provides indirect evidence for this recommendation. Caron et al. [388] conducted a case‐crossover study in middle‐aged patients (aged 45–64 years, *n* = 60,703), suggesting that anticoagulation prophylaxis may need to be extended up to 12 weeks postoperatively. For patients on preoperative antiplatelet therapy, individualized discontinuation based on bleeding risk is required. Meta‐analyses and cohort studies [382, 389, 390] indicate that low molecular weight heparin is safe and effective in older patients with lung cancer, although large‐scale RCT validation is lacking.

#### 3.3.4 Clinical Question 25: How to Prevent Postoperative Incisional Infection and Pneumothorax?

##### 3.3.4.1 Recommendation 48

Open surgical incision (1B), lack of preoperative prophylactic antibiotic use (1B), and T2DM (2C) are risk factors for postoperative incisional infection following lung cancer surgery.

##### 3.3.4.2 Recommendation 49

Risk factors for postoperative pneumothorax/prolonged air leak following lung cancer surgery include smoking history/failure to quit smoking preoperatively (2C), preoperative pulmonary dysfunction/COPD history (1B), absence of preoperative respiratory rehabilitation (2C), open surgical incision (2C), non‐utilization of fissureless lobectomy techniques (2C), and intraoperative non‐use of sealants for parenchymal wound coverage (1B).

##### 3.3.4.3 Rationale and Evidence Summary

Recommendation 48 is based on a systematic review conducted by the guideline working group on the association between surgical approach, diabetes status, and postoperative incisional infection in lung cancer surgery. A meta‐analysis of 9 retrospective cohort studies [391, 392, 393, 394, 395, 396, 397, 398, 399] showed that video‐assisted thoracoscopic surgery (VATS) reduced the risk of postoperative incisional infection compared to open surgery. A meta‐analysis of 8 prospective RCTs [400, 401, 402, 403, 404, 405, 406, 407] showed that preoperative prophylactic topical antibiotic use reduced postoperative incisional infection risk (RR = 0.61; 95% CI, 0.42–0.87). A meta‐analysis of two retrospective cohort studies [408, 409] showed that preoperative diabetes was associated with an increased postoperative incisional infection risk (RR = 8.62; 95% CI, 4.38–16.98). A meta‐analysis of five retrospective cohort studies [410, 411, 412, 413, 414] showed no significant difference in the incidence of postoperative incisional infection between lobectomy and segmentectomy.

The guideline working group conducted a systematic review on the associations between smoking history, preoperative pulmonary function, preoperative PR, surgical approach, surgical technique, intraoperative sealing of lung tissue wounds, and the occurrence of postoperative pneumothorax/persistent air leak in patients undergoing lung cancer surgery. A meta‐analysis of six retrospective cohort studies [415, 416, 417, 418, 419, 420] showed that a history of smoking was associated with an increased risk of postoperative pneumothorax/persistent air leak (RR = 2.00; 95% CI, 1.32–3.04). Analysis of two cohort studies [417, 421] indicated that poor preoperative pulmonary function was a risk factor for postoperative air leak. Analysis of two RCTs [422, 423] showed that the absence of preoperative PR was significantly associated with a higher incidence of postoperative persistent air leak. A meta‐analysis of five studies [395, 396, 397, 421, 424] showed that minimally invasive surgical approaches were associated with a reduced incidence of postoperative persistent air leak. A meta‐analysis of six cohort studies [425, 426, 427, 428, 429, 430] showed that fissure management techniques during lobectomy significantly reduced the incidence of postoperative persistent air leak (RR = 0.33; 95% CI, 0.18–0.60). A meta‐analysis of 8 RCTs [431, 432, 433, 434, 435, 436, 437, 438] showed that intraoperative use of sealing materials significantly reduced the incidence of postoperative pneumothorax/delayed air leak (RR = 0.36; 95% CI, 0.26–0.49).

#### 3.3.5 Clinical Question 26: How to Prevent Postoperative Urinary Tract Infection and Urinary Retention?

##### 3.3.5.1 Recommendation 50

When administering spinal/epidural anesthesia or postoperative analgesia, sufentanil is preferred over morphine. Early and progressive ambulation after surgery is recommended to prevent postoperative urinary retention (1A).

##### 3.3.5.2 Recommendation 51

Indwelling urinary catheterization duration should be minimized (≤24 h) to prevent and control catheter‐associated urinary tract infection (1B).

##### 3.3.5.3 Rationale and Evidence Summary

A systematic review [439] confirmed that replacing morphine with sufentanil in anesthesia protocols significantly reduces urinary retention risk, whereas postoperative non‐pharmacological interventions, such as progressive ambulation, reduce the risk by 65%. Two RCTs [440, 441] showed that ultrasound assessment of bladder volume is recommended when patients are unable to void at 4 h postoperatively. A systematic review [439] supports the use of suprapubic warm packs or warm gauze compresses to facilitate voiding in patients diagnosed with urinary retention. Systematic reviews and a Cochrane systematic review [442, 443] demonstrate that a shorter duration of indwelling urinary catheterization reduces urinary tract infections. A systematic review of 99 studies [444] also confirmed that early catheter removal (≤24 h) reduces catheter‐associated urinary tract infections and dysuria but increases the risk of recatheterization by 81%. Another systematic review [445] suggested that antiseptics, such as chlorhexidine or povidone‐iodine, may reduce infection rates, whereas the effectiveness of sterile technique remains controversial. Clinically, the risk of recatheterization should be balanced. It is recommended that healthcare institutions select antiseptics based on available resources to achieve potential benefits [443, 445].

#### 3.3.6 Clinical Question 27: How to Manage Postoperative Gastrointestinal Dysfunction?

##### 3.3.6.1 Recommendation 52

Multimodal analgesia is recommended to reduce opioid use while ensuring adequate pain control, thereby decreasing the risk of postoperative ileus and constipation (2B).

##### 3.3.6.2 Recommendation 53

In the absence of contraindications, early mobilization and resumption of oral fluid/food intake are recommended as fundamental measures to promote postoperative gastrointestinal recovery and prevent ileus and constipation (1A). Traditional Chinese medicine interventions, such as acupuncture (2B) and electroacupuncture (2A), may be considered to enhance postoperative bowel motility and alleviate pain. Sham feeding is recommended as an adjunctive intervention (1B).

##### 3.3.6.3 Rationale and Evidence Summary

One RCT showed that multimodal analgesia reduces opioid consumption and decreases gastrointestinal reactions such as postoperative ileus and constipation [446]. A meta‐analysis of two RCTs demonstrated that alvimopan (12 mg) significantly shortens postoperative hospital stay and time to gastrointestinal recovery [447, 448]. Two RCTs [449, 450] showed that acupuncture significantly shortens time to first flatus, first fluid intake, and first defecation; it also significantly reduces pain scores during activity, the incidence of 24‐h abdominal distension, and the incidence of postoperative nausea and vomiting. Two systematic reviews and one meta‐analysis [451, 452, 453] support the use of sham feeding to promote gastrointestinal function recovery after colorectal surgery. Postoperative gum chewing directly stimulates gastric, duodenal, and rectal peristalsis; sugar‐free gum containing xylitol promotes gastrointestinal motility and has an osmotic laxative effect, facilitating postoperative gastrointestinal function recovery [451, 452, 453, 454]. A meta‐analysis of 10 RCTs showed that sham feeding shortened time to flatus, time to first defecation, and hospital stay, and reduced complication rates; however, no differences in gastrointestinal function were found in trials with early postoperative feeding (*n* = 282). Sham feeding is safe after colorectal surgery, and although improvements are limited, it may reduce hospital stay [452].

#### 3.3.7 Clinical Question 28: How to Early Identify and Manage Postoperative Arrhythmias?

##### 3.3.7.1 Recommendation 54

Systematic preoperative assessment of arrhythmia risk is recommended in patients of advanced age (≥65 years), with a history of cardiac disease, reduced FEV_1_ (<70% predicted), left atrial enlargement (≥40 mm), or elevated BNP/NT‐proBNP levels. In high‐risk patients, enhanced intraoperative and postoperative electrocardiographic monitoring should be implemented, and postoperative analgesia should be optimized to reduce the risk of arrhythmia (2C).

##### 3.3.7.2 Recommendation 55

Strict intraoperative blood pressure control and minimization of surgical duration are recommended. Postoperatively, precipitating factors such as hypoxemia and electrolyte disturbances should be promptly corrected. For new‐onset postoperative atrial fibrillation, low‐dose landiolol or amiodarone is recommended for ventricular rate control or conversion to sinus rhythm; amiodarone may also be used for the treatment of postoperative supraventricular tachyarrhythmias. Anticoagulation therapy is recommended for atrial fibrillation persisting for more than 48 h or in patients with increased stroke risk. For hemodynamically unstable ventricular tachycardia, immediate electrical cardioversion is indicated; for stable sustained ventricular tachycardia, amiodarone or lidocaine may be considered (2C).

##### 3.3.7.3 Rationale and Evidence Summary

This recommendation is supported by cohort studies, meta‐analyses, and expert consensus. Age ≥65 years is consistently associated with postoperative arrhythmia, mainly atrial fibrillation, after lung cancer surgery [455, 456, 457, 458, 459, 460, 461, 462, 463, 464, 465, 466]. Key risk factors include COPD, prior cardiac disease, left atrial enlargement, FEV_1_ < 70% predicted, elevated BNP/NT‐proBNP, thoracotomy or extensive lung resection, and mediastinal lymph node dissection [455, 456, 457, 458, 459, 460, 461, 462, 463, 464, 465, 466, 467, 468, 469, 470, 471, 472, 473, 474, 475]. Pulmonary infection, hypoxemia, intrapericardial surgery, and inadequate analgesia may further increase risk [462, 476, 477, 478, 479]. Low‐dose landiolol [480] and amiodarone [481] are effective for postoperative atrial fibrillation, and amiodarone is also useful for supraventricular arrhythmias without clear evidence of increased respiratory complication after extensive lung resection [482]. Preventive strategies should focus on intraoperative blood pressure control, minimizing operative time, and correcting postoperative hypoxemia and electrolyte abnormalities [483]. BNP/NT‐proBNP and left atrial diameter may assist in identifying high‐risk patients. Sex and smoking are not listed as definitive predictors because of inconsistent evidence; further prospective studies are needed to refine individualized risk models.

## Funding

This work was supported by the Noncommunicable Chronic Diseases‐National Science and Technology Major Project (2023ZD0501803).

## Conflicts of Interest

All authors declare no conflicts of interest.

## Supporting information




**Supporting Information File 1**: jebm70178‐sup‐0001‐SuppMat.docx

## Data Availability

The data that support the findings of this study are available from the corresponding author upon reasonable request.
