# Supplementary material for: Guideline for the Comprehensive Perioperative Management of Older Patients With Lung Cancer (2025)
Source: J Evid Based Med. 2026 Aug 11;19(3):e70178. doi: 10.1111/jebm.70178 (PMC13613263; doi:10.1111/jebm.70178)
Supplement: Supplementary file 1 [file JEBM-19-0-s001.docx]

Supplementary Materials

Table S1. Search concepts and representative search terms

| Key search domain | Representative search terms |
| --- | --- |
| Population | older adults, elderly, aged, geriatric patients, lung cancer, lung carcinoma, non-small cell lung cancer, NSCLC |
| Perioperative context | perioperative period, preoperative assessment, postoperative care, lung cancer surgery, surgical management |
| Comprehensive management | comprehensive management, multidisciplinary care, integrated care, geriatric assessment, comprehensive geriatric assessment |
| Geriatric syndromes | frailty, sarcopenia, delirium, cognitive impairment, malnutrition, falls, sleep disturbance, anxiety, depression, functional decline |
| Comorbidities | diabetes mellitus, hypertension, coronary artery disease, chronic heart failure, atrial fibrillation, arrhythmia, chronic obstructive pulmonary disease, chronic kidney disease, osteoporosis, anemia |
| Complications | postoperative pulmonary complications, respiratory failure, postoperative infection, surgical site infection, pneumothorax, hemothorax, stroke, acute kidney injury, deep vein thrombosis, urinary retention, intestinal obstruction, constipation |
| Intervention and prognosis | early detection, risk assessment, treatment, intervention, rehabilitation, nursing care, prognosis, adverse events, length of hospital stay |

NSCLC, non-small cell lung cancer. The listed terms are representative examples. Database-specific searches were adapted using controlled vocabulary and free-text terms where applicable.

Table S2. Key outcome domains and representative indicators

| Key outcome domain | Representative indicators |
| --- | --- |
| Geriatric syndromes | frailty score, delirium, malnutrition, sarcopenia, falls |
| Physical function | grip strength, walking speed, ADL/IADL score, time to first ambulation |
| Psychological and sleep outcomes | anxiety score, depression score, sleep disturbance score |
| Symptom burden | pain score, oxygenation index |
| Cardiovascular outcomes | perioperative major adverse cardiovascular events, arrhythmia-related events |
| Pulmonary outcomes | postoperative pulmonary complications, respiratory failure, pneumothorax, hemothorax |
| Metabolic and renal outcomes | glycemic control achievement, hypoglycemic events, postoperative acute kidney injury |
| Hematologic and hepatic outcomes | hemoglobin target achievement, postoperative liver function deterioration |
| Thrombotic and bleeding outcomes | deep vein thrombosis, anticoagulant-related bleeding events |
| Surgical recovery outcomes | incision healing time, surgical site infection, time to first flatus or bowel movement, duration of urinary catheterization |
| Safety and healthcare utilization | adverse events, postoperative length of hospital stay |

ADL, activities of daily living; IADL, instrumental activities of daily living. The listed indicators are representative examples grouped by clinically relevant outcome domains.

Table S3. Appraisal approaches by evidence type and study design

| Evidence type or study design | Appraisal tool or approach |
| --- | --- |
| Systematic reviews | AMSTAR 2 |
| Randomized controlled trials | ROBUST |
| Non-randomized studies | ROBINS-I |
| Diagnostic accuracy studies | QUADAS-2 |
| Cohort studies | Newcastle–Ottawa Scale |
| Case-control studies | Newcastle–Ottawa Scale |
| Cross-sectional studies | AHRQ methodology checklist |
| Clinical practice guidelines | AGREE II, if applicable |

Table S4. Summary of Clinical Questions and Recommendations for Comprehensive Perioperative Management of Older Patients with Lung Cancer

| **Clinical Question** | **Recommendations** |
| --- | --- |
| **1. How to conduct preoperative frailty assessment and management?** | **Recommendation 1:** Routine frailty screening is recommended for patients aged ≥65 years undergoing lung cancer surgery (1A). The Geriatric 8 (G8) scale is suggested as the initial screening tool (2A). Patients with positive G8 screening (score ≤14) should undergo CGA (1B). Based on the assessment results, a stratified management approach is recommended: standard ERAS protocols for non-frail patients; a “prehabilitation + ERAS” model for prefrail patients; and individualized treatment through multidisciplinary consultation for frail patients (1A). |
| **2. How to assess and manage delirium risk and implement effective interventions?** | **Recommendation 2:** Timely identification of potential delirium is recommended for older patients with lung cancer during the perioperative period (1A). Assessment tools such as CAM or CAM- ICU may be used (1B), with an assessment frequency of at least once daily for a minimum of three days (2C). Preventive measures should be implemented for high-risk older patients (Table 1). Delirium prevention should primarily focus on multimodal non-pharmacological interventions (1B). Routine use of antipsychotic medications for prophylaxis is not recommended (1B). Short-term, low-dose, symptom-directed use may be considered only when non-pharmacological measures prove ineffective and significant agitation or safety risks are present (2C). |
| **3. How to assess the risk of perioperative malnutrition in older patients with lung cancer?** | **Recommendation 3:** NRS 2002 and/or MNA/MNA-SF are recommended for nutritional risk screening; the PG-SGA and/or GLIM criteria are recommended for the diagnosis and severity grading of malnutrition (2C).  **Recommendation 4:** For patients at risk of dysphagia, swallowing function screening should be initiated as early as possible (2C). The EAT-10 is recommended as the initial screening tool. For patients with positive screening results or at high risk, the V-VST is recommended for bedside assessment. Additionally, the Kubota Water Swallow Test, SSA, RSST, or GUSS can be used as supplementary tools (GPS). For patients with suspected severe dysphagia or recurrent aspiration, consultation with rehabilitation medicine specialists or swallowing therapists is recommended, with consideration of FEES or VFSS (2D) to confirm the diagnosis and guide subsequent clinical interventions (2C). |
| **4. How to formulate perioperative nutritional intervention strategies?** | **Recommendation 5:** Nutritional interventions are recommended based on the principles of “early intervention, individualized management, and multidisciplinary collaboration.” Individualized nutritional goals should be established, with dynamic assessment and adjustment in conjunction with measures such as exercise rehabilitation (GPS). For older patients (≥75 years), those with multiple chronic conditions, or those with frailty, multidisciplinary nutritional management involving or led by the geriatrics department is recommended (1B). |
| **5. How to assess and manage perioperative sarcopenia in older patients with lung cancer?** | **Recommendation 6:** Routine preoperative screening and assessment of sarcopenia are recommended (1A). CT-based muscle mass evaluation methods are recommended, particularly using the lumbar 3 (L3) skeletal muscle index (L3MI = (L3 skeletal muscle cross-sectional area) / (height²)) as the diagnostic criterion for sarcopenia, combined with muscle function indicators (such as grip strength, gait speed, or the five-time sit-to-stand test) for comprehensive evaluation (2B). For patients diagnosed with sarcopenia, comprehensive management combining exercise intervention and nutritional support is recommended (1A). Preoperative rehabilitation should be incorporated into routine management pathways (1B). |
| **6. How to conduct a preoperative fall risk assessment?** | **Recommendation 7:** Dynamic fall risk assessment is recommended (1A). HFRM and MFS are recommended as screening tools (1B). |
| **7. How to implement early postoperative rehabilitation to prevent falls?** | **Recommendation 8:** CGA (1A) and exercise intervention (1A) are recommended. For patients with fall risk or rehabilitation needs, personalized rehabilitation plans should be developed (2C), and comprehensive rehabilitation management centered on multifactorial intervention should be implemented in the early postoperative period (2B). |
| **8. How to conduct pain assessment and management?** | **Recommendation 9:** Comprehensive pain management throughout the entire perioperative period is recommended. Preoperative pain education is recommended (1C). Intraoperative use of local anesthetics for thoracic paravertebral nerve block, intercostal nerve block, or incisional infiltration aids in preventive analgesia (1B). Postoperatively, recommend daily dynamic assessment of resting and cough-related pain using standardized scales during hospitalization (2C). PCIA is recommended postoperatively (1C), with scheduled and as-needed analgesia, primarily using non-opioid analgesics, supplemented with opioids when necessary (1B). |
| **9. How to intervene in postoperative sleep disturbance?** | **Recommendation 10:** It is recommended to conduct baseline screening using the PSQI preoperatively, and dynamic assessment using RCSQ or NRS postoperatively. Intervention for postoperative sleep disturbance should be initiated when RCSQ <50 or NRS <5 (1B).  **Recommendation 11:** For patients with a preoperative PSQI ≥5 and postoperative RCSQ <50 or NRS <5, targeted interventions should be implemented based on the main influencing factors, including perioperative CBT delivered by MDT and multimodal analgesia management centered on combined SGB and PCIA during surgery (1B). |
| **10. How to assess ADL/IADL and set postoperative rehabilitation goals?** | **Recommendation 12:** It is recommended to assess ADL/IADL using the Katz-15 scale (2D). Postoperative rehabilitation goals should be established following the principle of being “achievable, quantifiable, evaluable, and time-bound”. Patients should be encouraged to actively participate in goal setting (GPS). |
| **11.** **How to assess and intervene for anxiety and depression?** | **Recommendation 13:** It is recommended to prioritize the use of the GDS-15 (or GDS-11 for Chinese-speaking patients) for depression assessment (1B). The GAI is recommended for anxiety screening, with threshold adjustment appropriate to the characteristics of the lung cancer population (2B).  **Recommendation 14:** For patients aged ≥65 years undergoing thoracoscopic radical resection for lung cancer, after excluding contraindications and conducting individualized assessment, consider perioperative low-dose esketamine administration, to reduce postoperative depression and anxiety scores. Mental status should be closely monitored during use, with vigilance for drug-related adverse reactions (2B).  **Recommendation 15:** Non-pharmacological interventions are recommended as the foundational strategy. CBT is effective in alleviating anxiety symptoms (1B) and shows a potential favorable trend in improving depression scores (2C). Additionally, a multidisciplinary integrated care model is recommended (1B) to promote comprehensive patient recovery. |
| **12. How to assess the risk of major perioperative cardiovascular events?** | **Recommendation 16:** Systematic preoperative assessment of medical history and cardiovascular risk factors is recommended, combined with clinical risk scoring tools and functional status evaluation to complete preliminary risk stratification (2B).  **Recommendation 17:** For patients aged ≥65 years or with prior history of chronic cardiovascular disease, comprehensive perioperative cardiovascular risk assessment is required, with dynamic monitoring of cardiac biomarkers and electrocardiogram within 72 hours postoperatively (1B).  **Recommendation 18:** For patients presenting with new-onset dyspnea, suspected heart failure, or high-risk factors including significant valvular disease, arrhythmia, or pulmonary hypertension, as well as those aged ≥75 years, transthoracic echocardiography and coronary CT angiography should be considered to clarify cardiac function and structural abnormalities. Specific protocols should be determined through shared decision-making between MDT and patient to establish optimal surgical timing (1B).  **Recommendation 19:** For patients with recent or worsening unstable acute coronary syndrome, hemodynamic instability, uncontrolled heart failure, or severe ventricular arrhythmias, elective surgery should be postponed in favor of prioritized cardiovascular optimization, with MDT management when indicated (1B). |
| **13. How to optimize perioperative medication management in patients with hypertension, coronary artery disease, chronic heart failure, and atrial fibrillation?** | **Recommendation 20:** Patients on long-term β-blockers (1B), CCBs (2C), or diuretics (2C) for blood pressure control should continue these medications throughout the perioperative period, with close monitoring of heart rate, blood pressure, electrolytes, and electrocardiographic activity. Angiotensin receptor-neprilysin inhibitors, ACEIs and ARBs should be withheld 24 hours prior to surgery. In hemodynamically stable patients, these medications can be resumed on the second postoperative day (1B). The use of α-blockers for perioperative blood pressure control is not recommended (2C).  **Recommendation 21:** In patients with coronary heart disease receiving single antiplatelet therapy for primary prevention should discontinue the medication. Whether to discontinue for secondary prevention requires evaluation by an MDT. Aspirin should be discontinued for ≥7 days, clopidogrel for ≥5 days, and ticagrelor for ≥3 days before surgery (1B). Surgery is not recommended for patients on DAPT (1A). If surgery is necessary, it is advisable to postpone the surgery until at least 6 weeks after PCI and discontinue one of the antiplatelet agents (2C).  **Recommendation 22:** For patients receiving NOACs, discontinuation 48 hours preoperatively is recommended, with resumption 48-72 hours postoperatively (1B). For patients with atrial fibrillation on warfarin, it should be discontinued at least 5 days before surgery, with management stratified based on CHA₂DS₂-VASc score. Heparin bridging is not recommended for patients without a high thromboembolic risk. The preoperative INR should be maintained below 1.5, and anticoagulation should be resumed 48-72 hours postoperatively (1B). For patients at a high thromboembolic risk, heparin bridging may be considered, with anticoagulation resuming 48-72 hours postoperatively. The decision regarding bridging therapy should arise from an MDT discussion, with enhanced the prevention of the perioperative bleeding risks (2C).  **Recommendation 23:** For older lung cancer patients with cardiac dysfunction, surgical decision and perioperative management plans should be collaboratively developed through MDT discussion (2C). |
| **14. How to assess the perioperative risk in patients with COPD?** | **Recommendation 24:** COPD is an important risk factor for surgery in older patients with lung cancer, particularly those aged ≥75 years (1B). ppo-FEV_1_ and ppo-TLCO serve as crucial parameters for evaluating perioperative risk (1B). CPET is recommended, and if the decline in SpO₂ exceeds 15%, surgical indications should be carefully evaluated (1B). Quantitative CT can aid in assessing perioperative risk in patients with COPD (2B). |
| **15. How to develop preoperative and postoperative pulmonary rehabilitation strategies for patients with COPD?** | **Recommendation 25:** Perioperative PR is recommended for older patients with lung cancer and comorbid COPD. PR should be initiated 2-4 weeks prior to surgery. The preoperative PR program should include respiratory function training, high-intensity exercise training, nutritional management, and psychological education. Weekly monitoring of pulmonary function, exercise tolerance, and other relevant indicators is essential to guide program adjustments. Postoperative rehabilitation should begin as early as possible, with early rehabilitation initiated on the first postoperative day. This structured approach should progress sequentially through the acute phase, recovery phase, discharge transition phase, and long-term maintenance phase (1A). |
| **16. How to establish glycemic control targets and management protocols for patients with comorbid diabetes?** | **Recommendation 26:** A comprehensive evaluation of the patient’s diabetes history should be conducted, including the measurement of FBG and HbA1c to assess glycemic control status and identify any diabetic complications before surgery. Continuous glycemic monitoring during surgery is recommended to guide adjustments in insulin dosage and administration. Postoperative FBG should be maintained below 10 mmol/L with dynamic monitoring to reduce the risk of infection (2C). |
| **17. How to manage medications in patients with comorbid chronic kidney disease (CKD)?** | **Recommendation 27:** For older patients with lung cancer and comorbid CKD and CVD, ACEIs are preferred for blood pressure control, while ARBs may serve as an alternative. The combination of ACEIs and ARBs should be avoided (1B).  **Recommendation 28:** Lipid management is indicated for all older patients with lung cancer and comorbid CKD and CVD, with statins being the first-line therapy. If low-density lipoprotein cholesterol targets are not met or if statins are not tolerated, cholesterol absorption inhibitors or PCSK9 inhibitors may be used either in combination or as alternatives (1B).  **Recommendation 29:** For older lung cancer patients with comorbid T2DM and CKD, SGLT2is are recommended, although they should be discontinued on the day of surgery. If glycemic targets are not achieved or SGLT2is are not tolerated, GLP-1 receptor agonists may be considered. The dosage of metformin should be adjusted based on renal function: contraindicated when the eGFR <30 mL/min/1.73 m²; not recommended as initial therapy when the eGFR <45 mL/min/1.73 m², and dose reduction is necessary for patients already receiving it (1B). |
| **18. How to assess, monitor, and correct patients with anemia?** | **Recommendation 30:** It is recommended to complete universal screening for anemia and etiological classification as early as possible preoperatively. Early identification and targeted intervention can reduce perioperative risks and improve clinical outcomes (1A).  **Recommendation 31:** For patients with iron deficiency anemia, intravenous iron is the preferred treatment. Oral iron may also be considered if the preoperative window allows sufficient time. For non-iron deficiency anemia, management should be individualized based on the specific underlying etiology. Perioperative blood transfusions should follow a restrictive threshold strategy (1B).  **Recommendation 32:** For patients with significant perioperative blood loss, limited time for correction, or inadequate response to iron therapy, an MDT should evaluate the initiation of ESAs. After excluding contraindications, short-term ESAs may be administered in conjunction with iron supplementation, with dynamic assessment of thromboembolic risk and individualized treatment under close monitoring (2B).  **Recommendation 33:** Throughout the perioperative period, hemoglobin and iron metabolism parameters should be dynamically monitored, with reassessments occurring on postoperative day 3 and prior to discharge. It is recommended to integrate anemia management into the thoracic surgery ERAS pathway and MDT framework to standardize implementation and ensure systematic follow-up (1B). |
| **19. How to monitor liver function and manage medication for patients with liver dysfunction?** | **Recommendation 34:** For older patients with lung cancer and concomitant hepatic impairment, liver function should be assessed preoperatively, and INR and liver reserve should be measured simultaneously. Patients shall be classified using the Child-Pugh or MELD scores, and perioperative medication should be adjusted accordingly (2C). |
| **20. How to assess and manage the risk of stroke?** | **Recommendation 35:** It is recommended to prevent venous thrombosis in older patients, physically inactive patients, patients with a heavy tumor burden, and those undergoing thoracotomy for lung cancer during the perioperative period. Routine monitoring of serum D-dimer levels should be conducted. If the level is significantly elevated, the risk of thrombosis and stroke is evaluated. After ruling out contraindications for bleeding, prophylactic anticoagulation should be considered (2C).  **Recommendation 36:** The frequency of brain MRI screening is determined based on tumor staging, molecular subtyping, and disease progression. For patients at high risk of brain metastasis, the evaluation frequency should be increased for early detection of brain metastasis or cerebrovascular events (2C).  **Recommendation 37:** For acute ischemic stroke with appropriate indications and no contraindications, mechanical thrombectomy for reperfusion therapy is preferred. This is particularly suitable for patients with unstable surgical wounds, active tumors, or those requiring continued anticoagulation, as it can safely and rapidly restore blood flow and preserve neurological function (2D).  **Recommendation 38:** Pay attention to the blood uric acid level. For patients with persistently low levels (<4 mg/dL), optimize their metabolic and nutritional status (2D). |
| **21. How to manage perioperative polypharmacy?** | **Recommendation 39:** Periodic medication reviews are recommended (1B) to reduce inappropriate medication use and improve medication adherence through interventions such as prescription simplification (1B), patient education (1C), and medication nursing (1C). |
| **22. How to prevent and early identify postoperative pulmonary complications?** | **Recommendation 40:** It is recommended to implement CGA combined with pulmonary function stratification. Use the ARISCAT score to assess the risk of PPCs and intervene based on the risk level. For high-risk patients, multi-modal pre-rehabilitation should be initiated 4-6 weeks before surgery (1A).  **Recommendation 41:** Use protective lung ventilation and restrictive/targeted fluid management, continuously monitor respiratory mechanics, and reduce ventilation-associated lung injury and acute respiratory distress syndrome (1B).  **Recommendation 42:** The I COUGH strategy is recommended for postoperative pulmonary rehabilitation, using multimodal or regional analgesia to ensure deep breathing and effective coughing, combined with nutritional and electrolyte optimization, to reduce the risk of atelectasis, infection, and hypoxia (2A).  **Recommendation 43:** Conduct intensive monitoring 48-72 hours after surgery. Use standardized criteria such as the European Perioperative Clinical Outcome or the Melbourne Group Scale to determine PPCs. Establish a continuous care pathway and audit metrics through collaboration among thoracic surgery, anesthesiology, respiratory/rehabilitation, geriatrics, and nutrition teams (1B). |
| **23. What are the procedures for the early identification and management of myocardial infarction/heart failure?** | **Recommendation 44:** Comprehensive cardiac assessment is recommended. Cardiac biomarkers should be monitored preoperatively and within 48-72 hours postoperatively. Cardiovascular risk stratification should be performed, and cardiac imaging should be selected individually to enable early identification of MI and heart failure (1B).  **Recommendation 45:** Individualized selection of interventional or surgical treatment versus conservative pharmacological management is recommended for older patients based on the type of perioperative MI and the risk associated with coronary revascularization (1B). In patients with chronic heart failure, continuation of the pre-existing treatment regimen is recommended; in those with acute heart failure, immediate pharmacological and non-pharmacological interventions should be initiated (1C). An MDT collaborative model is recommended (1B). |
| **24. Caprini score for deep vein thrombosis and timing of anticoagulation** | **Recommendation 46:** The Caprini Risk Assessment Model is recommended for evaluating the risk of VTE (1B), and stratified prevention strategies should be implemented based on the risk score. For high-risk patients (score≥5), pharmacological prophylaxis combined with mechanical prophylaxis is recommended (2C).  **Recommendation 47:** Anticoagulant medications should be discontinued 12-24 hours prior to surgery. Postoperatively, anticoagulation therapy should be resumed within 12-24 hours based on bleeding risk assessment, and extended prophylaxis up to 4 weeks post-surgery should be considered (1B). Low molecular weight heparin is the preferred pharmacological agent for VTE prophylaxis (2C). |
| **25. How to prevent postoperative incisional infection, hemothorax, and pneumothorax?** | **Recommendation 48:** Open surgical incision (1B), lack of preoperative prophylactic antibiotic use (1B), and type 2 diabetes mellitus (2C) are risk factors for postoperative incisional infection following lung cancer surgery.  **Recommendation 49:** Risk factors for postoperative pneumothorax/prolonged air leak following lung cancer surgery include: smoking history/failure to quit smoking preoperatively (2C), preoperative pulmonary dysfunction/COPD history (1B), absence of preoperative respiratory rehabilitation (2C), open surgical incision (2C), non-utilization of fissure-less lobectomy techniques (2C), and intraoperative non-use of sealants for parenchymal wound coverage (1B). |
| **26. How to prevent postoperative urinary tract infection and urinary retention?** | **Recommendation 50:** When administering spinal/epidural anesthesia or postoperative analgesia, sufentanil is preferred over morphine. Early and progressive ambulation after surgery is recommended to prevent postoperative urinary retention (1A).  **Recommendation 51:** Indwelling urinary catheterization duration should be minimized (≤24 hours) to prevent and control catheter-associated urinary tract infection (1B). |
| **27. How to manage postoperative gastrointestinal dysfunction?** | **Recommendation 52:** Multimodal analgesia is recommended to reduce opioid use while ensuring adequate pain control, thereby decreasing the risk of postoperative ileus and constipation (2B).  **Recommendation 53:** In the absence of contraindications, early mobilization and resumption of oral fluid/food intake are recommended as fundamental measures to promote postoperative gastrointestinal recovery and prevent ileus and constipation (1A). Traditional Chinese medicine interventions, such as acupuncture (2B) and electroacupuncture (2A), may be considered to enhance postoperative bowel motility and alleviate pain. Sham feeding is recommended as an adjunctive intervention (1B). |
| **28. How to early identify and manage postoperative arrhythmias?** | **Recommendation 54:** Systematic preoperative assessment of arrhythmia risk is recommended in patients of advanced age (≥65 years), with a history of cardiac disease, reduced FEV₁ (<70% predicted), left atrial enlargement (≥40 mm), or elevated BNP/NT-proBNP levels. In high-risk patients, enhanced intraoperative and postoperative electrocardiographic monitoring should be implemented, and postoperative analgesia should be optimized to reduce the risk of arrhythmia (2C).  **Recommendation 55:** Strict intraoperative blood pressure control and minimization of surgical duration are recommended. Postoperatively, precipitating factors such as hypoxemia and electrolyte disturbances should be promptly corrected. For new-onset postoperative atrial fibrillation, low-dose landiolol or amiodarone is recommended for ventricular rate control or conversion to sinus rhythm; amiodarone may also be used for the treatment of postoperative supraventricular tachyarrhythmias. Anticoagulation therapy is recommended for atrial fibrillation persisting for more than 48 hours or in patients with increased stroke risk. For hemodynamically unstable ventricular tachycardia, immediate electrical cardioversion is indicated; for stable sustained ventricular tachycardia, amiodarone or lidocaine may be considered (2C). |

CGA, Comprehensive Geriatric Assessment; ERAS, Enhanced Recovery After Surgery; CAM, Confusion Assessment Method; CAM-ICU, Confusion Assessment Method-Intensive Care Unit; NRS, Numerical Rating Scale; MNA, Mini Nutritional Assessment; MNA-SF, Mini-Nutritional Assessment Short-Form; GLIM, Global Leadership Initiative on Malnutrition; V-VST, Volume-Viscosity Swallow Test; WST, Water Swallow Tes; SSA, Standardized Swallowing Assessment; RSST, Repetitive Saliva Swallowing Test; GUSS, Gugging Swallowing Screen; FEES, Fiberoptic Endoscopic Evaluation of Swallowing; VFSS, Video Fluoroscopic Swallowing Study; GPS, HFRM, Hendrich Ⅱ Fall Risk Assessment Model; MFS, Morse Fall Scale; VRS, Verbal Rating Scale; PCIA, Patients-Controlled Intravenous Analgesia; PSQI, Pittsburgh Sleep Quality Index; RCSQ, Richards Campbell Sleep Questionnaire; PSQI, Pittsburgh Sleep Quality Index; CBT, Cognitive Behavioral Therapy; SGB, Stellate Ganglion Block; ADL, Activities of Daily Living; IADL, Instrumental Activities of Daily Living; GDS-15, Geriatric Depression Scale-15; GAI, Geriatric Anxiety Inventory; CT, Computed Tomography; CCBs, Calcium Channel Blockers; ARNIs, Angiotensin Receptor-Neprilysin Inhibitors; ACEIs, Angiotensin-Converting Enzyme Inhibitors; ARBs, Angiotensin Receptor Blockers; MDT, Multi-Disciplinary Treatment; DAPT, Dual Antiplatelet Therapy; PCI, Percutaneous Coronary Intervention; NOACs, Novel Oral Anticoagulant; INR, International Normalized Ratio; TIA, Transient Ischemic Attack; COPD, Chronic Obstructive Pulmonary Disease; PR, Pulmonary Rehabilitation; FBG, Fasting Blood Glucose; HbA1c, Hemoglobin A1c; CKD, Chronic Kidney Disease; CVD, Cardiovascular Diseases; ACE, Angiotensin-Converting Enzyme; LDL-C, Low-Density Lipoprotein Cholesterol; PCSK9, Proprotein Convertase Subtilisin/Kexin type 9; T2DM, Type 2 Diabetes Mellitus, SGLT2i, Sodium-dependent Glucose Transporter 2 inhibitor; GLP-1, Glucagon-Like Peptide-1; eGFR, Estimated Glomerular Filtration Rate; ESAs, Erythropoiesis Stimulating Agents; MELD, Model for End-Stage Liver Disease; MRI, Magnetic Resonance Imaging; ARISCAT, Assess Respiratory Risk in Surgical Patients in Catalonia; PPCs, Postoperative Pulmonary Complications; ARDS, Acute Respiratory Distress Syndrome; EPCO, European Perioperative Clinical Outcome; PMI, Perioperative Myocardial Infarction; VTE, Venous Thromboembolism; FEV₁, Forced Expiratory Volume in One Second; LAD, Left Atrial Anteroposterior Dimension; BNP, B-type Natriuretic Peptide; NT-proBNP, N-terminal pro-B-type natriuretic peptide

Supplementary Material A. Members of the Working Group for the Guideline

**Steering Committee:** Lunxu Liu (Department of Thoracic Surgery, West China Hospital, Sichuan University), Birong Dong (National Clinical Research Center for Geriatrics, West China Hospital, Sichuan University), Ning Ge (The Center of Gerontology and Geriatrics, West China Hospital, Sichuan University)

**Secretariat Group:** Daiping Li (National Clinical Research Center for Geriatric, West China Hospital, Sichuan University), Yang Wang (National Clinical Research Center for Geriatric, West China Hospital, Sichuan University), Zhongli Yang (National Clinical Research Center for Geriatric, West China Hospital, Sichuan University), Yixiao Chen (National Clinical Research Center for Geriatric, West China Hospital, Sichuan University)

**Consensus Expert Group (in alphabetical order by surname):** Guo Chen (Department of Anesthesiology, West China Hospital, Sichuan University), Huanwen Chen (Department of Thoracic Surgery, The First Affiliated Hospital of Chongqing Medical University), Lixia Chen (Department of Rehabilitation Medicine, Peking Union Medical College Hospital, Chinese Academy of Medical Sciences), Yuanda Cheng (Department of Thoracic Surgery, Xiangya Hospital, Central South University), Birong Dong (National Clinical Research Center for Geriatric Diseases, West China Hospital, Sichuan University), Langli Gao (The Center of Gerontology and Geriatrics, West China Hospital, Sichuan University), Ning Ge (The Center of Gerontology and Geriatrics, West China Hospital, Sichuan University), Qiukui Hao (The Center of Gerontology and Geriatrics, West China Hospital, Sichuan University), Peng Jiao (Department of Thoracic Surgery, Beijing Hospital, National Center for Gerontology, Institute of Geriatric Medicine，Chinese Academy of Medical Sciences), Runsen Jin (Department of Thoracic Surgery, Ruijin Hospital, Shanghai Jiao Tong University School of Medicine), Xiaofan Jing (Department of Clinical Nutrition, West China Hospital, Sichuan University), Hecheng Li (Department of Thoracic Surgery, Ruijin Hospital, Shanghai Jiao Tong University School of Medicine), Jing Li (Clinical Epidemiology and Evidence-Based Medicine Center, West China Hospital, Sichuan University), Shuo Li (Department of Thoracic Surgery, The First Affiliated Hospital of Xi'an Jiaotong University), Xin Li (Department of Lung Cancer Surgery, Center of Thoracic Surgery, Tianjin Medical University General Hospital), Hu Liao (Department of Thoracic Surgery, West China Hospital, Sichuan University), Peng Lin (Department of Thoracic Surgery, Sun Yat-sen University Cancer Center), Chengwu Liu (Department of Thoracic Surgery, West China Hospital, Sichuan University), Yongming Liu (Department of Geriatrics, The First Hospital of Lanzhou University), Lina Ma (Department of Geriatrics, Xuanwu Hospital Capital Medical University), Lin Ma (Department of Thoracic Surgery, West China Hospital, Sichuan University), Mao Mao (Department of Rehabilitation Medicine, The First Affiliated Hospital with Nanjing Medical University), Jiandong Mei (Department of Thoracic Surgery, West China Hospital, Sichuan University), Ben Niu (Department of Geriatrics,The First People's Hospital of Yunnan Province, The Affiliated Hospital of Kunming University of Science and Technology), Chunfeng Pan (Department of Thoracic Surgery, The First Affiliated Hospital of Nanjing Medical University), Qiang Pu (Department of Thoracic Surgery, West China Hospital, Sichuan University), Le Shen (Department of Anesthesiology, Peking Union Medical College Hospital, Chinese Academy of Medical Sciences and Peking Union Medical College), Jiajun Wang (Department of Thoracic Surgery, The First Hospital of China Medical University), Jun Wang (Department of Thoracic Surgery, The First Affiliated Hospital of Nanjing Medical University), Yanfu Wang (Department of Geriatrics, The First Affiliated Hospital of Dalian Medical University), Shiyou Wei (Department of Thoracic Surgery, West China Hospital, Sichuan University), Quan Wei (Rehabilitation Medicine Center, West China Hospital, Sichuan University), Yang Xia (Department of Thoracic Surgery, The First Affiliated Hospital with Nanjing Medical University), Pengming Yu (Rehabilitation Medicine Center, West China Hospital, Sichuan University), Jirong Yue (The Center of Gerontology and Geriatrics, West China Hospital, Sichuan University), Chunfang Zhang (Department of Thoracic Surgery, Xiangya Hospital, Central South University), Guangjian Zhang (Department of Thoracic Surgery, The First Affiliated Hospital of Xi'an Jiaotong University), Haiyan Zhang (Department of Geriatrics, The First Hospital of China Medical University), Shuguang Zhang (Department of Thoracic Surgery, The First Hospital of China Medical University), Ju Zhu (Department of Thoracic Surgery, The First Affiliated Hospital of Chongqing Medical University)

**Evidence Evaluation Group (in alphabetical order by surname):** Lei Chen (Department of Thoracic Surgery, West China Hospital, Sichuan University), Nan Chen (Department of Thoracic Surgery, West China Hospital, Sichuan University), Miao Dai (Department of Geriatrics, Jiujiang No.1 People's Hospital), Xiang Ding (The Center of Gerontology and Geriatrics, West China Hospital, Sichuan University), Shuju Dong (Department of Geriatrics,The First People's Hospital of Yunnan Province, The Affiliated Hospital of Kunming University of Science and Technology), Xiaoyun Fan (Department of Geriatric Respiratory and Critical Care Medicine, The First Affiliated Hospital of Anhui Medical University), Fanyi Gan (Department of Thoracic Surgery, West China Hospital, Sichuan University), Lijuan Guan (Department of Geriatrics, Chengdu Fifth People's Hospital affiliated to Chengdu University of Traditional Chinese Medicine), Minzhang Guo (Department of Thoracic Surgery, West China Hospital, Sichuan University), Jianqi Hao (Department of Thoracic Surgery, West China Hospital, Sichuan University), Xinxin He (The Center of Gerontology and Geriatrics, West China Hospital, Sichuan University), Yuting Huang (Department of Geriatrics, Guangyuan Central Hospital), Chuan Li (Department of Thoracic Surgery, West China Hospital, Sichuan University), Daiping Li (National Clinical Research Center for Geriatric, West China Hospital, Sichuan University), Linlong Li (The Center of Gerontology and Geriatrics, West China Hospital, Sichuan University), Rui Liang (The Center of Gerontology and Geriatrics, West China Hospital, Sichuan University), Taiping Lin (The Center of Gerontology and Geriatrics, West China Hospital, Sichuan University), Tianjian Lu (Department of Thoracic Surgery, West China Hospital, Sichuan University), Linyu Luo (The Center of Gerontology and Geriatrics, West China Hospital, Sichuan University), Qing Ma (General Practice Ward, General Practice Medical Center, West China Hospital, Sichuan University), Zhenyi Niu (Department of Thoracic Surgery, West China Hospital, Sichuan University), Lin Su (The Center of Gerontology and Geriatrics, West China Hospital, Sichuan University), Jiantong Sun (Department of Thoracic Surgery, West China Hospital, Sichuan University), Xuelian Sun (National Clinical Research Center for Geriatric, West China Hospital, Sichuan University), Xiangping Tu (Department of Laboratory Medicine, West China Hospital, Sichuan University), Jiao Wang (Rehabilitation Medicine Center, West China Hospital, Sichuan University), Jiao Wang (National Clinical Research Center for Geriatric, West China Hospital, Sichuan University), Qian Wang (Health Management Center, West China Hospital, Sichuan University), Yang Wang (National Clinical Research Center for Geriatric, West China Hospital, Sichuan University), Zihuai Wang (Department of Thoracic Surgery, West China Hospital, Sichuan University), Liang Xia (Department of Thoracic Surgery, West China Hospital, Sichuan University), Ping Xu (Medical Library Knowledge Service Center, Sichuan University Library), Lin Xu (Rehabilitation Medicine Center, West China Hospital, Sichuan University), Lulu Xu (Department of Geriatrics, Chongqing General Hospital), Dan Yang (The Center of Gerontology and Geriatrics, West China Hospital, Sichuan University), Zhenyu Yang (Department of Thoracic Surgery, West China Hospital, Sichuan University), Lian Ye (Department of Respiratory and Critical Care Medicine, West China Hospital, Sichuan University), Tao Ying (The Center of Gerontology and Geriatrics, West China Hospital, Sichuan University), Yanpeng Zhang (Department of Thoracic Surgery, The First Affiliated Hospital of Xi'an Jiaotong University), Kejia Zhao (Department of Thoracic Surgery, West China Hospital, Sichuan University), Quan Zheng (Department of Thoracic Surgery, West China Hospital, Sichuan University), Ruihao Zhou (Department of Anesthesiology, West China Hospital, Sichuan University)

**External Review Expert Group (in alphabetical order by surname):** Xinyu Chen (Department of Geriatrics, Zhejiang Hospital), Lin Kang (Department of Geriatrics, Peking Union Medical College Hospital, Chinese Academy of Medical Sciences), Sheyu Li (Department of Endocrinology and Metabolism, Laboratory of Diabetes and Metabolism Research, West China Hospital, Sichuan University), Xiaohong Liu (Department of Geriatrics, Peking Union Medical College Hospital, Chinese Academy of Medical Sciences), Wei Xiong (Department of Geriatric Medicine and Special Medical Services, Southwest Hospital, Army Medical University), Qin Zhang (Department of Geriatrics, the First Affiliated Hospital, School of Medicine, Zhejiang University), Xun Zhang (Department of Thoracic Surgery, Tianjin Chest Hospital), Songbai Zheng (Department of Geriatrics, Huadong Hospital Affiliated to Fudan University)
